# Proteome-wide association studies identify biochemical modules associated with a wing-size phenotype in *Drosophila melanogaster*

**DOI:** 10.1038/ncomms12649

**Published:** 2016-09-01

**Authors:** Hirokazu Okada, H. Alexander Ebhardt, Sibylle Chantal Vonesch, Ruedi Aebersold, Ernst Hafen

**Affiliations:** 1 Institute of Molecular Systems Biology, ETH Zurich, Wolfgang Pauli Strasse 16, Zürich 8093, Switzerland; 2 Faculty of Science, University of Zurich, Zurich 8057, Switzerland

## Abstract

The manner by which genetic diversity within a population generates individual phenotypes is a fundamental question of biology. To advance the understanding of the genotype–phenotype relationships towards the level of biochemical processes, we perform a proteome-wide association study (PWAS) of a complex quantitative phenotype. We quantify the variation of wing imaginal disc proteomes in *Drosophila* genetic reference panel (DGRP) lines using SWATH mass spectrometry. In spite of the very large genetic variation (1/36 bp) between the lines, proteome variability is surprisingly small, indicating strong molecular resilience of protein expression patterns. Proteins associated with adult wing size form tight co-variation clusters that are enriched in fundamental biochemical processes. Wing size correlates with some basic metabolic functions, positively with glucose metabolism but negatively with mitochondrial respiration and not with ribosome biogenesis. Our study highlights the power of PWAS to filter functional variants from the large genetic variability in natural populations.

## Abstract (web summary)

How genetic diversity generates complex phenotypes along a continuum remains a fundamental question of biology. Here—applying the emerging SWATH proteomics technology—the authors describe a proteome wide association study (PWAS) of Drosophila wing size and identify functional protein clusters associated with this trait.

## Graphical abstract

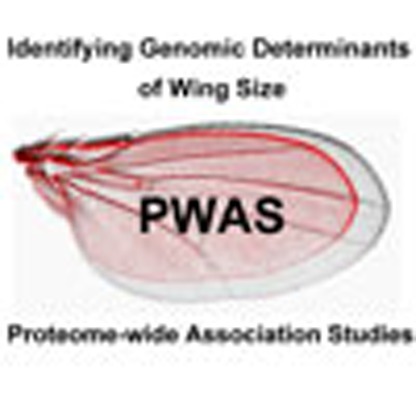

Single gene analyses by traditional forward and reverse genetics approaches in model organisms revealed evolutionarily conserved signalling pathways that control growth^1^,^2^,^3^,^4^,^5^,^6^. Yet, it is presently unknown whether these same pathways are also the major determinants of growth and size variation of individuals in natural populations. Previous studies did not provide insights into intra-species variability. Furthermore, previous studies neglected the fact that natural selection acts on phenotypes that, for the most part, are the product of complex interactions between genomes and the environment over time, and not the product of single genes. Genome-wide association studies (GWAS) correlate markers spread over entire genomes with phenotypes and have mapped many quantitative trait loci (QTLs) that affect natural variation in phenotypic traits^7^,^8^. The inbred lines of the *Drosophila* genetic reference panel (DGRP) provide a good model system for such association studies, as the inter-strain genetic diversity reflects that of a wild population^9^. Remarkably, the genomes of inbred lines generated from individuals of a single population exhibit ∼25-fold higher single-nucleotide polymorphism (SNP) diversity than is observed in a human population^9^,^10^,^11^. Furthermore, experiments with *Drosophila* can be performed under controlled environmental conditions, whereas it is difficult to account for environmental factors in human GWAS studies^12^,^13^,^14^. The mechanistic interpretation of GWAS results has been hampered by the fact that genomes contain coding, non-coding, functional and non-functional genetic variants that have accumulated over evolutionary time, and that are difficult to distinguish in association studies. In contrast, genetically determined variability in protein sequence or abundance has been shown to provide a more direct link between biochemical mechanisms and phenotypes^15^,^16^. We would therefore expect that variation at the level of proteins is more tightly associated with phenotypic variation than genomic variation.

## Results

### Tight control of protein abundance in wing discs

**Figure 1:**
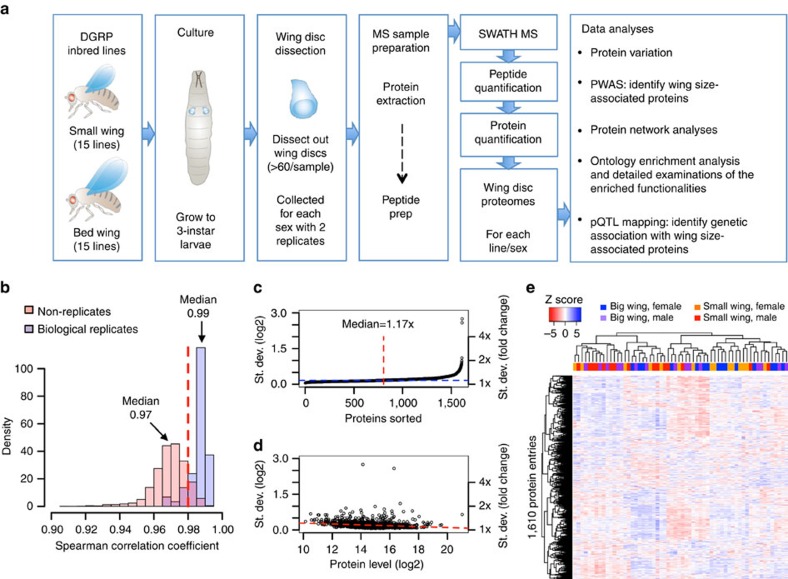
Experimental scheme and variation of wing disc proteins. (**a**) Flow of the experiments. Wing discs from wing-size-extreme *Drosophila* inbred lines were dissected and collected. SWATH-MS quantified wing disc proteomes for each line/sex, which were analysed to identify/characterize wing-size-associated proteins. (**b**) Reproducibility of the experiment. Pairwise Spearman's rank correlation coefficients between peptide levels showed higher correlations among biological replicates than among non-replicates. (**c**) Variation of protein levels; s.d. is plotted in an increasing manner. (**d**) Relationship between protein variation and protein abundance. Less abundant proteins show larger variations. (**e**) Cluster analysis of the proteome data matrix. Proteins (1,610 entries) and samples (30 lines × 2 sexes) are hierarchically clustered based on Spearman's correlations.

Here we used the complex phenotype ‘wing size' in *Drosophila melanogaster* to test whether functionally relevant variation is more readily detected at the proteome than the genome level. We chose the wing-size phenotype, because extensive single-gene analyses have been conducted, environmental influences can be controlled and because it can be precisely measured morphometrically. We used sequential, windowed acquisition of all theoretical masses (SWATH) mass spectrometry (SWATH-MS), a massively parallel and highly reproducible protein quantification technique^16^,^17^,^18^ to quantify 1,610 protein entries extracted from wing imaginal discs, the precursor tissue of the adult wing. To maximize the between-line size variation, we selected 30 lines with extreme wing-size phenotypes (15 with big wings and 15 with small wings) from the DGRP line collection (Fig. 1a). To account for the sex-dimorphic nature of wing size in *Drosophila*, we dissected and collected wing discs from third instar larvae separately for each sex. Biological duplicates were prepared for each line/sex, resulting in a total of 120 disc samples that were analysed by SWATH-MS. Computational analysis of the resulting data sets with the OpenSWATH software tool^19^ allowed us to identify and quantify 6,755 unique peptides in 119 samples. All alleles basically occur in the homozygous state within an inbred line and therefore a peptide containing polymorphic protein coding variation is either fully detected in samples with the reference sequence or completely undetected in samples in case of a coding variant. In the latter case, the protein level is determined based on the other constituent peptides that are not coding variants. Thus, our data do not contain measurements that might be inaccurate when a coding variation exists in the heterozygous state. Pairwise Spearman's rank correlation coefficients of peptide levels between biological replicates showed nearly perfect reproducibility (median 0.99) of quantification, whereas coefficients between non-replicates showed a left-shifted, distinct distribution (median 0.97), indicating larger variability between than within genotypes (Fig. 1b and Supplementary Fig. 1). We determined the levels of 1,610 protein entries as the mean of the constituent peptides that were fit for each line and sex using a linear model (see Methods and Supplementary Data 1). A fraction of the proteins had multiple entries (238 entries for 101 proteins), because they were identified as differently annotated sequence variants, and we therefore designated them using entry numbers (see Methods and Supplementary Data 2). We observed that 87% of the protein entries showed significant variation between lines or sexes (Supplementary Fig. 2) but, surprisingly, the median standard deviation (s.d.) in protein levels was only 17% (in fold change) in spite of the extensive genetic variability among lines (Fig. 1c). More abundant proteins tended to show slightly smaller variation, suggesting that more abundant proteins are less affected by genetic variation among lines (Fig. 1d). To obtain an overview over the entire data structure, we applied hierarchical clustering to proteins and samples (lines/sex) based on Spearman's rank correlations (Fig. 1e). Both big and small wing samples spread across the clusters, indicating similar overall structures of the proteomes between big and small wing discs. Overall, these data indicate that wing disc proteomes have an unexpectedly small variability in spite of the large inter-line genomic variability, suggesting a strong buffering capacity at the protein level.

### Proteome-wide association study

**Figure 2:**
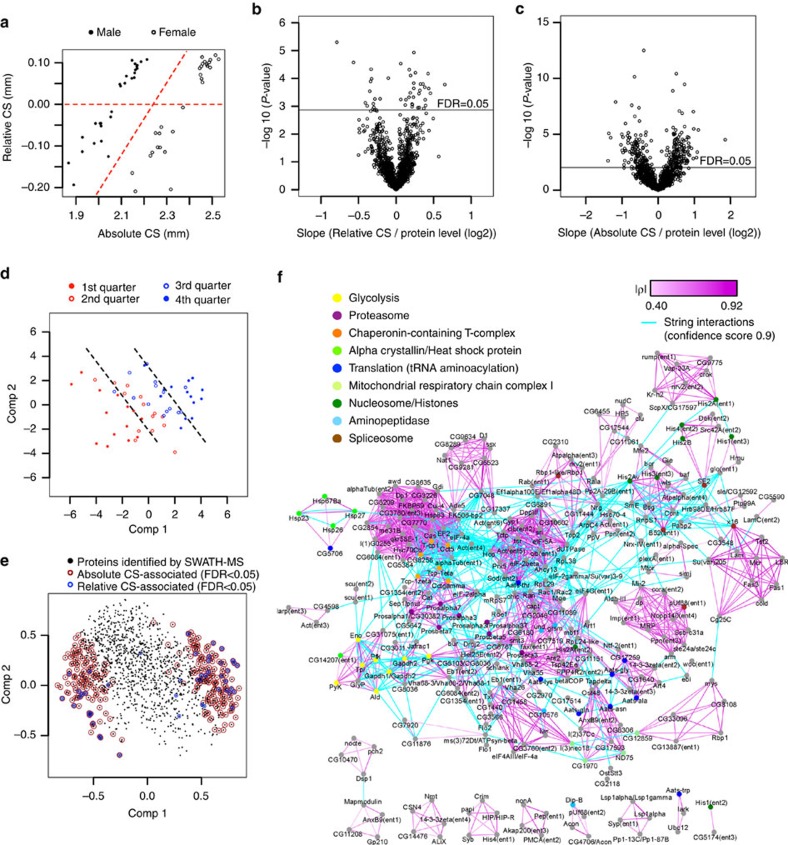
Protein network of wing-size-associated proteins. (**a**) CS of wings at adult age. Absolute CS and relative CS (adjusted for body size) were used as explanatory variables in PWAS. (**b**,**c**) Association of proteins with relative and absolute CSs, respectively. *P*-values are plotted against the slope of the fitted line. The horizontal line indicates 5% FDR threshold. (**d**) Score plot against the first two PLS components. Samples sorted by wing size into four groups are aligned along the components in an increasing manner. (**e**) Correlation loadings plot. Correlation between proteins and the PLS components are plotted. The wing-size-associated proteins are marked as indicated. (**f**) Protein network and functionality of the wing-size-associated proteins. Protein covariation modules were identified based on absolute Spearman's correlation (|*ρ*|>0.4, equivalent to *P*-value <0.001). Strength of connection is indicated by the tone of purple colour. Protein interactions (cyan edges) based on STRING database at the highest confidence (Score=0.9) were combined to construct a large wing-size-associated protein network. Enriched functionalities identified by David are indicated by node colours as indicated.

To establish an association between proteome abundance variation and phenotypic variation, we next performed a proteome-wide association study (PWAS). Specifically, we evaluated an association between the abundance distribution of each quantified protein and the phenotype wing size. We first defined the wing-size phenotype using centroid size (CS) that is a standard measure of the ‘size' of a shape in geometric morphometrics. We considered two wing CSs: absolute CS that is principally proportional to wing area and suitable to analyse sex-dependent difference of wing size (Supplementary Fig. 3), and relative CS that is adjusted for body size using interocular distance (IOD) (see Methods and Supplementary Data 3). Relative CS classified our samples into 15 big and 13 small wing lines for each sex (Fig. 2a) (it is noteworthy that 2 small wing lines were removed for all following data analyses due to the unavailability of genotype information). For PWAS, the two variables absolute and relative CS were regressed on protein levels (see Methods). After multiple testing correction by the Benjamini–Hochberg method, 46 and 304 protein entries were identified to be associated with relative and absolute CSs, respectively, at a false discovery rate (FDR) of 5% (Fig. 2b,c and Supplementary Data 4). To visualize the wing-size-associated proteins in the whole proteome data set, we performed two different dimension-reduction methodologies: principal component analysis (PCA) and partial least squares (PLS). Although PCA better explained variation in the proteome, PLS was superior to PCA in capturing wing-size variation (Supplementary Fig. 4). For both wing-size measures, the first two PLS components explained >70% of variation in size. We therefore plotted our samples against the two PLS components derived to explain absolute CS (Fig. 2d). Both components aligned the samples in an increasing order of wing size, confirming that they describe the wing-size variation well. Plotting of the correlation between proteins and the two PLS components revealed that the proteins associated with relative and absolute CS were mostly overlapping and mapped in the top-right region (for positive correlation to wing size) and the bottom-left region (for negative correlation) of the plot (Fig. 2e). These data indicate that ∼20% of the quantified proteins are associated with wing size and about one half correlates positively and the other half negatively.

### Wing-size-associated protein modules

To estimate functional connectivity of the variant proteins, we applied hierarchical clustering to the wing-size-associated proteins using Spearman correlation (*ρ*) as a similarity measure. We identified high-correlation modules by cutting off connections at |*ρ*|=0.4, which is equivalent to a *P*-value of 0.001. The protein modules were combined with protein interactions from the STRING10 database at the highest confidence criteria (Score=0.9), which led to the construction of a large wing-size-associated protein network (303 nodes connected with 1,560 edges) that consisted of most of the associated proteins (Fig. 2f and Supplementary Data 5). To identify functionalities embedded in the network, we performed Gene Ontology enrichment analysis. The functionalities enriched include glycolysis (*p*=1.4 × e^−14^), proteasome (*p*=2.1 × e^−12^), nucleosome/histones (*p*=3.0 × e^−13^) and mitochondrial respiratory chain complex I (*p*=7.3 × e^−7^). Strikingly, the proteins implicated in the cellular processes were mostly found enriched in specific modules, suggesting that the proteins in the same processes co-vary across lines.

**Figure 3:**
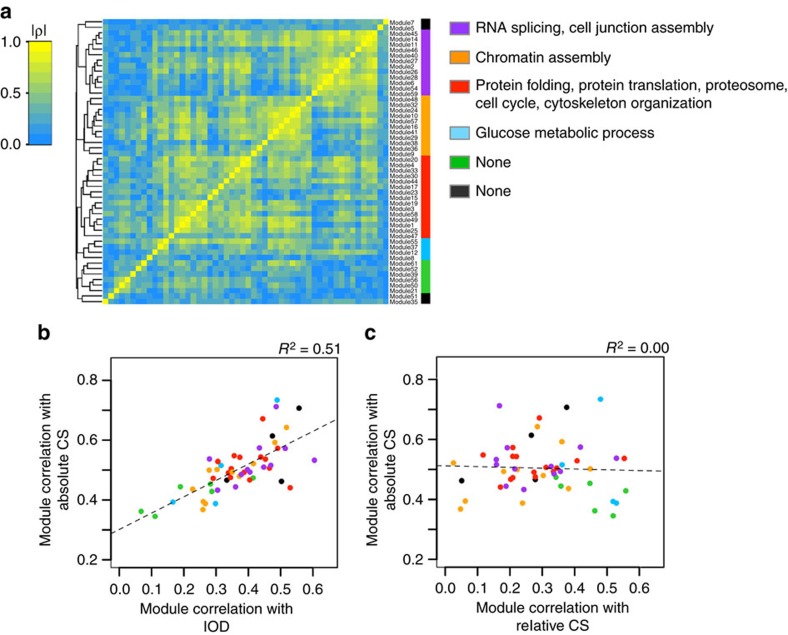
Protein module connectivity and correlation with size traits. (**a**) Higher-order clustering of protein modules. The modules were hierarchically clustered based on Spearman correlation (|*ρ*|) between the principal components of the individual modules. Functionalities enriched for the higher-order module clusters are shown. The significance for the enrichment was determined (<0.05) by Benjamin–Hochberg method. (**b**) Relationship between module correlations with absolute CS and IOD. Spearman's correlation between the principal components of the modules and size traits are plotted. The fitted line and the *r*^2^ from linear regression are shown. (**c**) Relationship between module correlations with absolute and relative CSs.

To investigate on inter-module relationship, we applied hierarchical clustering to the protein modules. The similarity between the modules was defined as the Spearman correlation between the principal components of the individual modules. The higher-order clustering revealed five big module clusters. Distinct cellular functionalities were attributed to four of these clusters (Fig. 3a). To investigate module-level association with size traits, correlations between modules and size traits were analysed (Supplementary Data 6). Module correlation with wing size (absolute CS) showed a linear relationship with that with IOD (Fig. 3b). This indicates that the size of different body parts correlates in a similar way with biochemical processes, suggesting a similar mechanism of size control in the whole body. Interestingly, lower correlations were seen with the green- and blue-coloured module clusters that were enriched with proteins implicated in glucose metabolism. Absolute CS correlated well with all the modules but relative CS showed an uneven distribution of correlation with modules (Fig. 3c). High correlation with relative CS was prominently observed with the green- and blue-coloured module clusters. This suggests that the purple (RNA splicing, cell junction assembly)/orange (chromatin assembly)/red (protein folding and translation, proteosome, cell cycle and cytoskeletal organization) module clusters correlate with the body size in general and the green/blue (glucose metabolic process) module clusters exhibit a relatively specific correlation with wing size.

### Discrete correlations of metabolic processes with wing size

**Figure 4:**
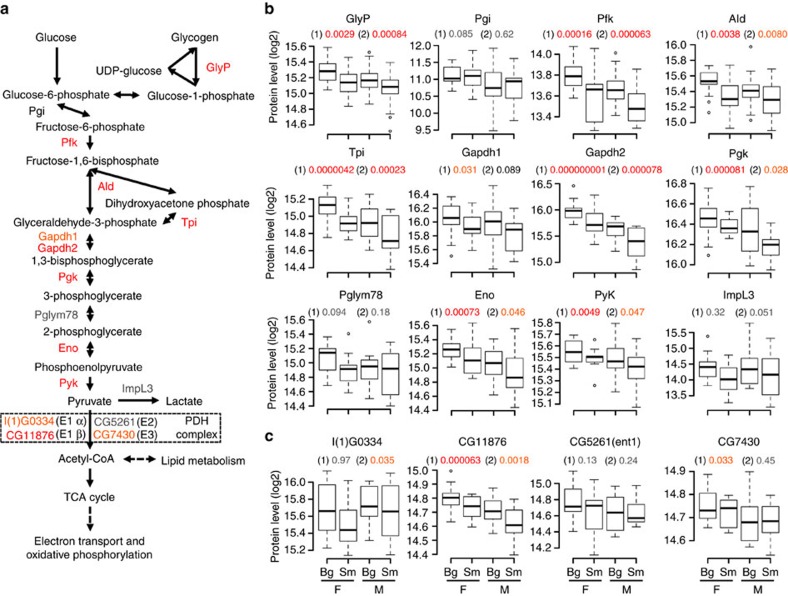
Systemic association of glucose metabolism with wing size. (**a**) Pathway map for glucose metabolism. Proteins detected by SWATH-MS are shown. Proteins in red indicates association with either of absolute or relative CS at 5% FDR. Proteins in orange indicates association at nominal *P*-value <0.05. (**b**) Glycolytic protein levels plotted against wing size for each sex. *P*-values are shown for association with absolute CS (1) and relative CS (2). Significance levels of association are indicated by colour of *P*-values as in **a**. Bg, big wing samples; F, female; M, male; Sm, small wing samples. (**c**) Subunit proteins from pyruvate dehydrogenase (PDH) complex are plotted against wing size for each sex.

To draw mechanistic insights from the process-level associations identified by PWAS, we examined the variation of all glycolytic proteins in the proteome. Glycolysis comprises ten enzymatic steps through which glucose is decomposed into pyruvate with the generation of ATP and NADH. SWATH-MS identified ten enzymes from glycolysis and two enzymes responsible for glycogen breakdown and lactate fermentation (Fig. 4a). Surprisingly, the protein levels of all these enzymes showed positive correlation to wing size in both sexes (Fig. 4b). This observation was confirmed statistically, as eight enzymes associated with absolute or relative CS at 5% FDR and one enzyme at a nominal *P*-value <0.05. In addition, phosphofructokinase, the key enzyme in the control of glycolytic flux, exhibited one of the strongest associations with wing size. Three out of four subunits of the pyruvate dehydrogenase complex, including the rate-limiting E1 subunits that convert pyruvate to acetyl-CoA, were also positively correlated with size (Fig. 4c). These observations support an association between larger wing size and an increased use of glycolysis.

**Figure 5:**
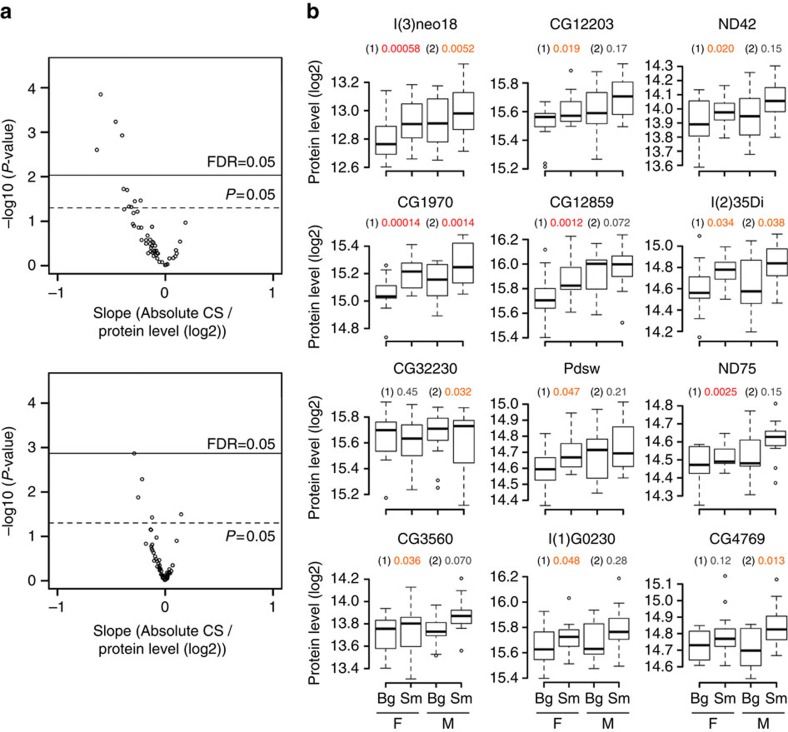
System-level negative association of mitochondrial respiration with wing size. (**a**) Negatively biased slope distribution of mitochondrial respiratory chain complex proteins. The *P*-values obtained in PWAS for absolute and relative CSs are plotted against slopes fitted in the model. The horizontal lines indicate significance thresholds as indicated. (**b**) Levels of mitochondrial respiratory chain complex proteins associated with wing size are plotted against wing size for each sex. *P*-values are shown for association with absolute CS (1) and relative CS (2). Significance levels of association are indicated by colour of *P*-values as in Fig. 4b. Bg, big wing samples; F, female; M, male; Sm, small wing samples.

Following up on this observation, we further investigated downstream processes of glucose metabolism with respect to a correlation with size: the enzymes responsible for the tricarboxylic acid cycle that oxidizes acetyl-CoA to CO_2_, to produce NADH, FADH_2_ and ATP mostly did not pass the significance threshold (Supplementary Fig. 5a,b) but showed a weak positive correlation to wing size (Supplementary Fig. 5c). SWATH-MS detected 53 proteins from mitochondrial respiratory chain complexes that use NADH/FADH_2_ to produce ATP (Supplementary Table 1). Surprisingly, these proteins contrastingly showed a strong negative correlation at the systemic level (Fig. 5a). Individual inspection of the wing-size-associated respiratory chain complex proteins confirmed their negative correlation to wing size in both sexes (Fig. 5b). This systematic negative correlation is specific to respiratory chain complex proteins in mitochondria, as other mitochondrial proteins such as the enzymes of the tricarboxylic acid cycle (Supplementary Fig. 5c) and ribosomal proteins (Supplementary Fig. 6) did not show negative correlation. The target-of-rapamycin (TOR) signalling pathway positively controls cellular and organismal growth^1^,^4^,^20^. Cytosolic ribosomal proteins that are targets of the TOR signalling did not show a bias to wing size (Supplementary Fig. 7). Previous studies reported that TOR signalling regulated expression of most of the genes both in glycolysis^21^ and in mitochondrial oxidation^22^, a finding that is inconsistent with our results. The activity of upstream regulators of growth is in many cases mediated by posttranslational modifications of pathway components (such as phosphorylation), which we did not detect in the current study, and therefore association studies of posttranslational modification together with the study of protein abundance is desirable to determine whether the activity of TOR signalling is related to these systemic associations. Together, these results implicate that larger wing tissues use more glucose metabolism and less mitochondrial respiratory metabolism, which resembles the characteristics of highly proliferative cancer cells (Warburg effect) on a smaller scale.

Furthermore, we detected a systemic association between histone protein levels and wing size (Fig. 2f and Supplementary Fig. 8), whereas other nuclear proteins such as nuclear pore complexes and spliceosomes showed no such trend (Supplementary Fig. 9). Cellular histone protein levels are thought to be constant as long as the cells have the same length of genomic DNA^23^. We therefore investigated whether this applies to our case. We first measured cell sizes in the adult wing among the lines of the smallest and biggest wings (Supplementary Fig. 10). Interestingly, cell size was different between sexes but invariant within each sex, except for the two smallest wing lines. These results indicate that wing-size variation within each sex is mainly determined by cell number (Supplementary Fig. 11). Assuming that cell size in the adult wing is proportional to cell size in the wing disc, we estimated relative histone protein abundance per cell in the extreme samples (see Methods). The approximate histone levels per cell do not exhibit systemic correlation with wing size (Supplementary Fig. 12), suggesting that the negative correlation of histone levels observed in our proteome mostly reflects the cell size variation among the samples. Thus, these analyses suggest that our data are in line with the ‘constant' cellular histone protein levels.

### Genetic association of wing-size-associated proteins

**Figure 6:**
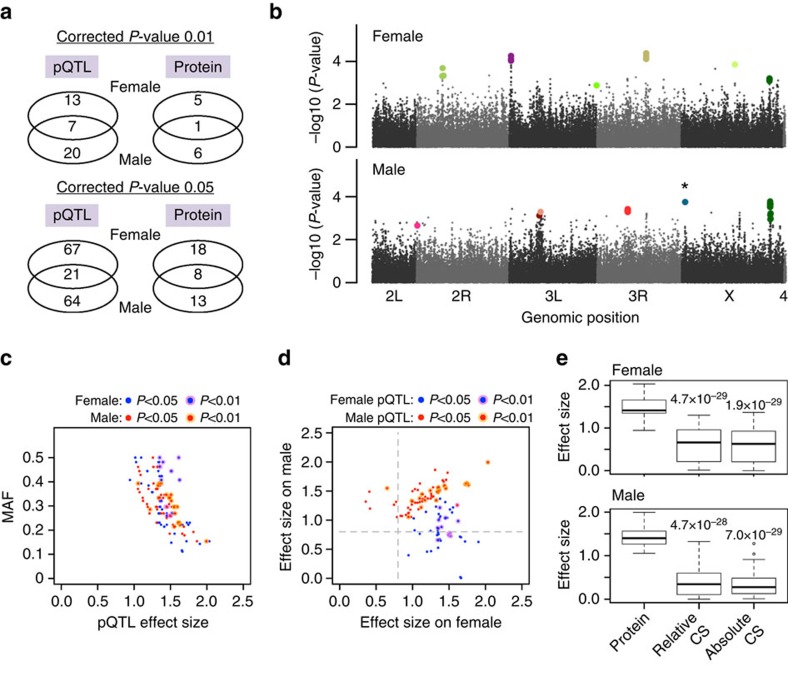
Genetic association of wing-size-associated proteins. (**a**) *cis*-pQTL mapping for the wing-size-associated proteins. The numbers of pQTLs and protein entries identified for each sex at different significance thresholds are depicted using Venn diagrams. (**b**) Manhattan plots. *P*-values for protein/*cis*-SNP association tests are plotted with black dots along the genetic coordinates. pQTLs at a corrected *P*-value threshold of 0.01 are shown as coloured dots, which *cis*-associate to 6 (7) protein entries in female (male). *A pQTL shared by two protein entries. (**c**) Distribution of effect size and minor allele frequency (MAF) for pQTLs. Effect size is measured by the standardized difference in the two means between genotypes. The sex and significance threshold of pQTLs are indicated. (**d**) Effect size of pQTLs on the protein of the opposite sex is plotted. The dashed lines indicate effect size at 0.8. Values >0.8 are classified as large by Cohen's criteria. (**e**) Comparison of effect sizes between on protein and on wing size. The statistical significance was evaluated using Wilcoxon rank-sum test.

Despite the high genetic variation in *Drosophila*^9^,^10^,^11^, we observed a strong buffering capacity at the protein level, indicating a remarkable robustness of cellular and biochemical processes against genomic variation. Such molecular resilience may have evolved to allow for genetic variation to accumulate, which may then be beneficial under changing environmental conditions. To investigate genetic association to wing-size-associated proteins, we performed protein QTL (pQTL) mapping. To identify potential *cis*-regulatory variants, we tested association of protein levels to SNPs located within ±10 kb of the gene region with minor allele frequency>10%. We applied mapping using the Kruskal–Wallis test in each sex separately, as protein levels were significantly different between sexes for many proteins (Supplementary Fig. 2a). Multiple testing correction was performed through permutation, as previously described^10^,^24^,^25^. At a corrected *P*-value threshold of 0.01 (0.05), we detected 11 (39) proteins with at least one pQTL significant in either sex (Fig. 6a,b and Supplementary Data 7). Owing to the limited sample size, the number of pQTLs identified in the study is relatively small. Most of the pQTLs were found to be sex specific, which is consistent with a previous study of expression QTL mapping in *Drosophila*^10^. We found that all pQTLs have large effect sizes (>0.8 in Cohen's criteria) for both sexes (Fig. 6c and Supplementary Data 8), indicating that the protein levels are clearly distinct between SNP variants (Supplementary Figs 13 and 14). The majority of pQTLs in one sex also showed large effect sizes in the opposite sex (> 0.8) (Fig. 6d), suggesting that the pQTLs basically exert their effect on both sexes. As the proteins for which we mapped pQTLs are associated with wing size, we investigated the effect of the pQTLs on wing size (Fig. 6e). The effect sizes for wing size were, however, significantly smaller than those for proteins, indicating that the effect of pQTLs attenuates from protein levels towards the downstream phenotypic level.

## Discussion

Studies of the molecular intermediates between genotype and phenotype have been thought critical to advance the mechanistic understanding of the genotype–phenotype relationships^26^. Previous reports indicate that protein levels weakly correlate with messenger RNA levels^27^,^28^,^29^,^30^ such that studies of the two molecular levels would provide distinct structures for the association map. Recent advances in proteomics technologies, specifically the ability of SWATH-MS to accurately quantify consistent sets consisting of hundreds to thousands of proteins across extended sample cohorts now make PWAS technically feasible. We have performed, to our knowledge, the first PWAS on a complex quantitative trait, which revealed that basic biochemical processes are associated with wing-size variation. Our results indicate that protein levels in the biochemical processes tightly associate with phenotypes, probably because phenotypic traits are formed through the biochemical processes that are performed by proteins. We found that biochemical processes are enriched in specific protein co-variation clusters. Similar observations were reported in previous human and mouse studies^15^,^25^. The process-level detection of association in our study stems from this co-variation feature of the proteins within the same processes.

We have observed molecular resilience at the protein level that buffers a very large genetic variability in *Drosophila* towards stable phenotypes on which selective pressure acts. This buffering capacity may indicate that variation in protein abundance is more functionally relevant than genetic variation and proteins provide a functional filter on genomic variation. Even though our study shows a striking co-variation of components of different biochemical pathways involved in growth, it also highlights present limitations of the technique. Our previous shotgun MS study of whole fly bodies identified ∼9,000 proteins using a combination of diversified samples, multi-dimensional biochemical fractionation and the repeated experimental loops and the data from hundreds of liquid chromatography–tandem MS (LC–MS/MS) analyses were cumulated^31^. In contrast, in the present study proteins were extracted from a highly specialized tissue at a specific developmental time point and analysed in a single injection without extensive proteome fractionation. In addition, we were only able to use a limited amount of samples for each fly line due to the time-consuming dissection process. More than 8,500 wing discs were dissected for 120 MS samples resulting in about 38 μg of protein extract per sample on average. Therefore, the number of proteins identified was limited. Our protein detection is still largely focusing on relatively abundant proteins and we miss the information on many proteins with regulatory roles usually expressed at lower levels. In general, for any analytical method including SWATH-MS, the uncertainty of measurement for signals close to the limit of detection is larger than for signals with a high signal-to-noise ratio. To address this issue, in our study only peptides that were reliably quantified in more than 96 samples were used for further analyses (see Methods for more detail). The protein levels for 95% of the wing-size-associated proteins are more than eight times higher than the minimum abundance among the proteins quantified in the study. This focus on signals with robust signal-to-noise ratios increased the accuracy of quantification for the wing-size-associated proteins, thus assuring the robustness of our findings on the wing-size-based association map and protein network.

Our study highlights the discrete association of metabolic processes with size. The findings implicate the higher use of glycolysis in bigger tissues, which seems natural when tissues are to grow more. It is, however, striking that oxidative phosphorylation negatively correlates with wing size and ribosome biogenesis shows no systemic difference between big and small wing lines. We recently performed a GWAS study on size traits using 143 DGRP lines^32^. The QTL mapping on wing size identified 111 QTLs spread throughout the fly genome that are located near/in 130 gene regions. The genes identified in the GWAS are mostly not canonical growth genes and RNA interference knockdown tests confirmed 33 genes to be novel growth regulators of wings. Out of the 130 genes, we detected 10 at the protein level in the current study and 3 proteins (CG3011, CG6084 and Gdi) were found to be associated with wing size at 5% FDR (Supplementary Table 9). CG3011 and CG6084 are both metabolic enzymes and were confirmed to modulate wing size.

This study has revealed systemic associations among genome, proteome and size traits in the *Drosophila* wing; however, the causal relationships of the associations remain to be determined. We demonstrate advantages and limitations of PWAS to uncover biochemical processes that correlate with a complex phenotype and thus advance the understanding of the black box lying between genotype and phenotype.

## Methods

### *Drosophila* culture and wing disc dissection

Flies were cultured at 25 °C under non-crowding conditions with food that contained 100 g of fresh yeast, 55 g of cornmeal, 10 g of wheat flour, 75 g of sugar and 8 g of bacto agar per litre medium. Third instar larvae wandering on the wall of the culture vial were transferred in ice-cold Hank's balanced salt solution, where wing discs were dissected under the microscope and collected separately for each sex in tubes containing ice-cold Hank's balanced salt solution buffer and kept at −80 °C until use. This process was repeated at different dates and cultures so that the total number of wing discs per line/sex/replicate became more than 60 (up to 110).

### Morphometrics of adult wings

Size measurements of adult wings were performed in our previous study^32^. The raw data of the size measurements are provided^32^. Briefly, 143 DGRP lines were set up in duplicate vials on the same day. After three generation of inbred crossing, F_3_ L1 larvae were distributed into three replicate vials, each containing 40 larvae. Owing to the different developmental timing among lines, the food for F3 larvae was prepared at different times among lines, which categorized lines into four groups by the food batches. The adult F_3_ flies were pooled from the three vials 1–2 days after eclosion and kept at −20 °C. Wings were taken and photographed under a VHX-1000 digital light microscope (KEYENCE). Morphometric measurements were extracted using WINGMACHINE^33^ and MATLAB (MATLAB version R2010b, The MathWorks Inc., Natick, MA) and the raw CS was calculated as the square root of the summed squared distances of 14 landmarks from the centre of the wing. IOD was measured as the distance from eye edge to eye edge.

Absolute CS was derived using a regression model (*CS*_raw_=*μ*+S+FB+GI+*ɛ*, where S denotes sex, FB denotes foodbatch and GI denotes genomic inversion). The distinct days of food preparation were reflected in the variable ‘foodbatch', with four levels representing food prepared according to the same recipe and procedure on four distinct days. The inversions In (2L)t and In (3R)Mo were coded as (0,1,2), depending on whether no, one or two inversions was present in the homozygous state. To make absolute CS to reflect the sex-dimorphic nature of wing size, absolute CS was defined by subtracting effects of foodbatch and inversion (but not sex) from the raw CS. Relative CS was defined as described^32^. Briefly, the raw CS was regressed (*CS*_raw_=*μ*+IOD+S+FB+*ɛ*, where IOD denotes the trait covariate) to define relative CS. IOD was used as a representative measure for body size. Inversions were not modelled, because the residual CS did not show correlation with any inversion. The residual *ɛ* obtained by this model was defined as relative CS. Analyses were performed in the R statistical environment (version 3.1.2) (http://www.r-project.org).

### MS sample preparation

The tubes containing wing discs were thawed and centrifuged at 6,000 r.p.m. for 2 min on table-top centrifuges. The supernatant was removed and the tissues were lysed by pipetting up and down in 100 μl of lysis buffer (6 M urea, 0.2% RapiGest (Waters) and 50 mM ammonium bicarbonate). The lysate was transferred into the next tube and wing discs were mixed and lysed. This was continued until the last tube, to have more than 60 wing discs dissolved in 100 μl lysis buffer. The combined lysate was sonicated for 10 min in water bath and the protein content was measured using a bicinchoninic acid assay (Thermo Scientific). The proteins were reduced with 10 mM dithiothreitol for 30 min at 60 °C in a shaker (750 r.p.m.) and, after cooling down, alkylated with 55 mM iodoacetamide for 1 h at room temperature, while shaking at 700 r.p.m. in the dark. The samples were diluted with 50 mM ammonium bicarbonate to be 1.5 M concentration of urea. After checking pH∼8.0, sequencing grade trypsin (Promega) was added to a substrate:enzyme ratio of 40:1 and incubated at 37 °C overnight on the shaker (550 r.p.m.). Although the samples were acidified (pH<3), tC18 columns (Sep-Pak Vac 1cc (100 mg), Waters) were pre-wet by 100% methanol and subsequently by 80% acetonitrile (ACN) and 0.1% trifluoroacetic acid (TFA) and equilibrated with 0.1% TFA. The acidified samples were then applied to the columns three times and the columns were washed with 0.1% TFA. Peptides were eluted with 1 ml of 50% ACN and 0.1% TFA, and dried by centrifugal evaporation. The peptides were resuspended in 0.1% formic acid and 5% ACN to be 0.3 μg μl^−1^ and kept at −20 °C until use.

### SWATH mass spectrometry

Relative peptide levels within each sample were determined in SWATH-MS: first, we analysed various wing disc samples by LC–MS/MS in shotgun mode and built a high-confidence reference spectral library^34^. Each sample was then analysed in SWATH mode^17^,^35^. From the resulting SWATH data set, which contains fragment ion spectra of all peptide ions in a user-determined retention time versus peptide ion mass-to-charge ratio, individual peak groups were extracted using OpenSWATH and the integrated area under the curve per peak group was summed up to obtain an intensity per peptide^19^. Each peak group consists of the chromatographic elution profile of a set of fragment ion signals that, collectively, uniquely identify a particular peptide. The summed single intensity (integrated peak area of the transition traces identifying a peptide) was used as a quantitative indicator for relative quantitative comparisons of the peptide in question across the samples. In more detail, SWATH-MS data were acquired using an AB Sciex 5600 TripleTOF mass spectrometer interfaced to an Eksigent NanoLC Ultra 2D Plus HPLC system. Samples were chromatographed using a 120-min gradient from 2 to 35% (buffer A 0.1% (v/v) formic acid, 2% (v/v) ACN, buffer B 0.1% (v/v) formic acid and 90% (v/v) ACN) after direct injection onto a 20 cm PicoFrit emitter (New Objective) packed to 20 cm with Magic C18 AQ 3 μm 200 Å stationary phase. For SWATH-MS-based experiments, the mass spectrometer was operated in SWATH mode using a precursor isolation width of 26 *m/z* covering the precursor mass range of 400–1,200 *m/z*. This *m/z* setting effectively resulted in 32 isolation windows (400–425, 424–450, … 1,174–1,200) SWATH MS2 ion traces were detected from 100–2,000 *m/z*. The collision energy for each window was determined according to the calculation for a charge 2^+^ ion centred on the window with a spread of 15 eV. An accumulation time of 100 ms was used for all fragment-ion scans in high-sensitivity mode and for the survey scans in high-resolution mode acquired at the beginning of each cycle, resulting in a duty cycle of ∼3.4 s^17^,^35^.

### Peptide quantification and reproducibility analysis

The reference library for targeted extraction of ion traces from SWATH files was generated by acquiring a representative peptide pool of all samples on the AB Sciex 5600 TripleTOF mass spectrometer interfaced with an Eksigent NanoLC Ultra 2D Plus HPLC system in data-dependent mode. LC settings were identical to SWATH-MS acquisition mode. MS1 spectra were collected in the range 360–1,460 *m/z* and the 20 most intense precursors with charge state 2–5 exceeding 250 counts per second were selected for fragmentation. MS2 spectra were collected in the range 50–2,000 *m/z* for 100 ms and, subsequently, the precursor ions were dynamically excluded from reselection for 20 s.

The acquired vendor-specific data files were converted into mzXML format. COMET and X!Tandem *in silico* search engines annotated peptide spectrum matches against FlyBase r5.52 protein database using Carbamidomethyl (Cys) as static modification and Oxidation (Met) as variable modification. Following the initial searches, the peptides were evaluated by PeptideProphet^36^. At 1% FDR, 1,685 proteins were identified in the pool of wing disc proteome. Based on these search results, the spectral library is generated for targeted SWATH extraction as described by Schubert *et al*.^34^. With the spectral library in hand, SWATH files are analysed using openSWATH^19^,^37^ implemented in the iPortal GUI^38^. Search results are stored in OpenBIS file server system^39^. Exact parameters of the informatics SWATH workflow are given in the Supplementary Note 1.

The peptide abundance was quantified using the area under the curves summing up co-eluting transitions from the SWATH-MS ion chromatograms, so that the abundance was considered to be a continuous variable. Therefore, the measurement did not suffer from high sampling variances at low abundance, which has been an issue when abundance is measured by count data such as spectral counting. However, as for any analytical method including SWATH-MS, the uncertainty of measurement for signals close to the limit of detection is larger than for signals with a high signal-to-noise ratio. To limit this effect on the associations determined in this study, peptides with small, unreliable signals close to the limit of detection were treated to have missing values, thereby avoiding inaccurate assertions of values to them. If a particular peptide was observed as missing values in more than 20% of SWATH-MS maps among samples, it was removed from further analyses. The net result of this filtering step was that we only considered peptides that were reliably quantified in more than 96 samples, which resulted in a set of 6,755 peptides that were used for the association studies.

The intensities for peptides were log_2_-transformed and the normalization from sample to sample was performed by median centralization. To evaluate reproducibility, Spearman's rank correlation coefficients were calculated in a pair-wise manner between samples. The distribution of the coefficients was compared between within replicates and within non-replicates.

### Protein annotation and quantification

In the study, 1,610 protein entries were quantified, each of which are different due to their distinct constituent peptides. The entries consist of 3 types as follows: (1) 1,248 entries uniquely represent each 1,248 protein; (2) 238 entries represent 101 proteins according to multiple entries for each protein. This happened, because each protein was identified by distinct sets of peptides among its variants. Thus, these proteins have multiple entries, which are designated with entry numbers. (3) The remaining protein entries (124 entries) represent protein isoforms. The isoforms share the same peptides and, therefore, are indistinguishable. They are designated by combining the names of the isoforms.

Model-based quantification of protein levels was performed using a linear model. For each protein entry, the MS intensities were regressed on MS features (peptides) and biological features (30 lines × 2 sexes), and error variances were estimated from biological replicates: MS peak intensity=MS feature+biological feature+*ɛ* (by replicates). Ninety-four per cent of the protein entries showed significant variation across biological features. Protein levels for each line/sex were calculated as the mean of the constituent peptides fitted by the model. Significance of protein variation for sexes and lines separately were evaluated by regressing protein levels on sex and line. *P*-value 0.05 was used as the significance threshold for each factor, revealing 762 protein entries significant for sex and 1,324 entries for line, and in combination, 1,394 entries (87%) in total. As most of the proteins exhibited significant variation, all the proteins quantified by MS were used in the data analyses. The size of protein variation was estimated using the s.d. across the samples.

### Proteome-wide association study

To identify proteins that are associated with wing size, we applied a linear model to each protein entry and evaluated the significance level of association. The model used for relative CS was: relative CS=sex+protein level (in log_2_ scale)+*ɛ*. The interaction between sex and protein was not significant for all proteins at 5% FDR. In the model for absolute CS, a simple regression was performed to identify proteins associated with the sex-dimorphic nature of wing size: absolute CS=protein level (in log_2_ scale)+*ɛ*. The correction for multiple testing was performed using Benjamini–Hochberg method. The FDR was estimated by p.adjust() function in R.

### Network and Gene Ontology enrichment analyses

PCA and PLS regression analyses were performed using pls package^40^ in R, in which 1,342 protein entries that contain no missing values were considered. To construct the wing-size-associated protein network, the wing-size-associated proteins identified by PWAS was hierarchically clustered using hclust() function in R with complete linkage, where the protein co-variation similarity was defined as the absolute value of Spearman's correlation coefficients (*ρ*). To identify the high co-variation modules, the connections were cut at |*ρ*| <0.4, equivalent to *P*-value 0.001. The protein interactions among the wing-size-associated proteins identified by STRING (version 10) (http://www.string-db.org) at the highest confidence level (Score=0.9) were then added to the co-variation modules. The network was visualized using the open-source platform Cytoscape (version 3.1.1) (http://www.cytoscape.org). The biological processes enriched for the wing-size-associated proteins were identified by DAVID (https://david.ncifcrf.gov/) using the functional classification tool. The higher-order module clusters were identified by performing another hierarchical clustering against the modules with multiple protein components using hclust() function in R with complete linkage. The module similarity was defined by the absolute value of Spearman's correlation coefficients (*ρ*) between the principal components of individual modules. The principal components were calculated using prcomp() function in R. Functionalities enriched for the higher-order module clusters were identified using DAVID with the significance threshold 0.05 by Benjamin–Hochberg method. Correlation between the modules and size traits was also defined by Spearman's correlation coefficients (|*ρ*|).

### Cell size measurement and histone protein levels per cell

To investigate cell size variation in the wing among the lines of the five smallest wings and four biggest wings, we first determined the cell number in the wing by counting the trichomes in a fixed 100 μm × 100 μm square area of the third posterior cell region in the ventral side of the wing. The cell number for each line/sex was defined as the mean of 15–20 flies. The single cell area was calculated as the defined area (10^4^ μm^2^) divided by the cell number in the square region. The total cell number in the whole wing was estimated as the whole wing area divided by the single cell area.

It has been suggested that histone levels are determined only by DNA length. If this were the case, histone levels are estimated lower in bigger cells, as protein levels were normalized to the whole protein content (by median centralization). Assume that there are two cells, a big cell (volume=2X) and a small cell (volume=1X), and both cells have the same level of histone protein (amount=Y). In this situation, our normalized protein levels become Y/2 for big cell and Y for small cell. We can convert the histone protein levels to relative protein levels per cell by multiplying the current protein levels by cell volumes:

Big cell: Y/2 × 2X–> XY

Small cell: Y × X–> XY

To apply this procedure, we need to estimate relative cell volumes of wing discs. We assumed that cell areas in the wing determined above approximate the cell area variation in wing discs. Cell volume was then calculated as (cell area)^3/2^. The relative histone protein levels per cell were finally obtained by transforming the current protein levels in log2 scale back to a linear scale and by multiplying them by the cell volumes. Thus, we compared the relative protein levels per cell among samples.

### pQTL mapping

Genotypes of the 28 lines for pQTL mapping were obtained from the DGRP Freeze 2 (http://dgrp2.gnets.ncsu.edu). The tests for association between wing-size-associated proteins and *cis*-SNPs were performed in R using Kruskal–Wallis test, applying for each sex separately. Only the *cis*-SNPs located within ±10 kb of the gene region with minor allele frequency>10% among 28 lines were considered. Multiple testing correction was performed through permutation as previously described^10^,^24^,^25^. We repeated each test for 10,000 permutations of protein expression in the whole proteome. For each permutation, the minimum *P*-value was recorded among all SNPs for each protein entry. A corrected *P*-value was calculated as the number of minimum *P*-values from the permutations that were smaller than the original *P*-value divided by the number of permutation. Effect size is defined as the mean difference between genotypes divided by the s.d. The effect size was calculated in each sex.

### Data availability

All the raw MS data including the spectral library of fly wing proteome and the OpenSWATH outputs are stored at Center for Computational Mass Spectrometry (http://proteomics.ucsd.edu) with MassIVE ID: MSV000079202 and MSV000079208. The additional data that support the findings of this study are available from the corresponding author upon request.

## Additional information

**How to cite this article:** Okada, H. *et al*. Proteome-wide association studies identify biochemical modules associated with a wing-size phenotype in *Drosophila melanogaster*. *Nat. Commun.* 7:12649 doi: 10.1038/ncomms12649 (2016).

## Supplementary Material

Supplementary Information Supplementary Figures 1-14, Supplementary Table 1 and Supplementary Note 1

Supplementary Data 1 Log2 protein levels for 1,610 protein entries among 60 wing disc samples

Supplementary Data 2 Protein annotation with FlyBase polypeptide ID

Supplementary Data 3 Wing phenotypic traits

Supplementary Data 4 PWAS (association between protein abundance and wing size)

Supplementary Data 5 Protein network of the wing size-associated proteins

Supplementary Data 6 Protein module correlation with size traits

Supplementary Data 7 pQTL mapping

Supplementary Data 8 Effect sizes of pQTLs

Supplementary Data 9 Proteins implicated in GWAS
