# Supplementary material for: Proteome-wide association studies identify biochemical modules associated with a wing-size phenotype in Drosophila melanogaster
Source: Nat Commun. 2016 Sep 1;7:12649. doi: 10.1038/ncomms12649 (PMC5025782; doi:10.1038/ncomms12649)
Supplement: Supplementary Information — Supplementary Figures 1-14, Supplementary Table 1 and Supplementary Note 1 [file ncomms12649-s1.pdf]

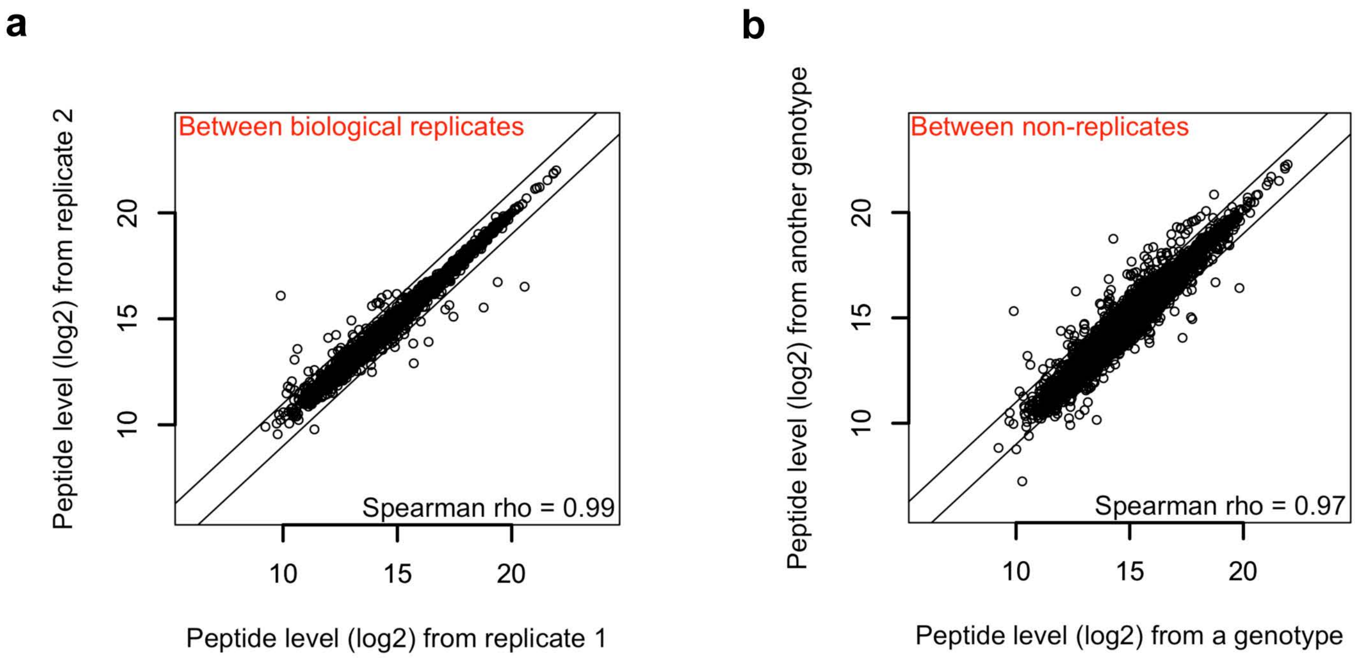

Supplementary Figure 1. Correlation comparison of peptide levels between replicates and non-replicates

a, Correlation between biological replicates. An example of xy-plot between peptides of biological replicates is shown. **b**, Correlation between non-replicates. An example of xy-plot between peptides of non-replicates is shown.

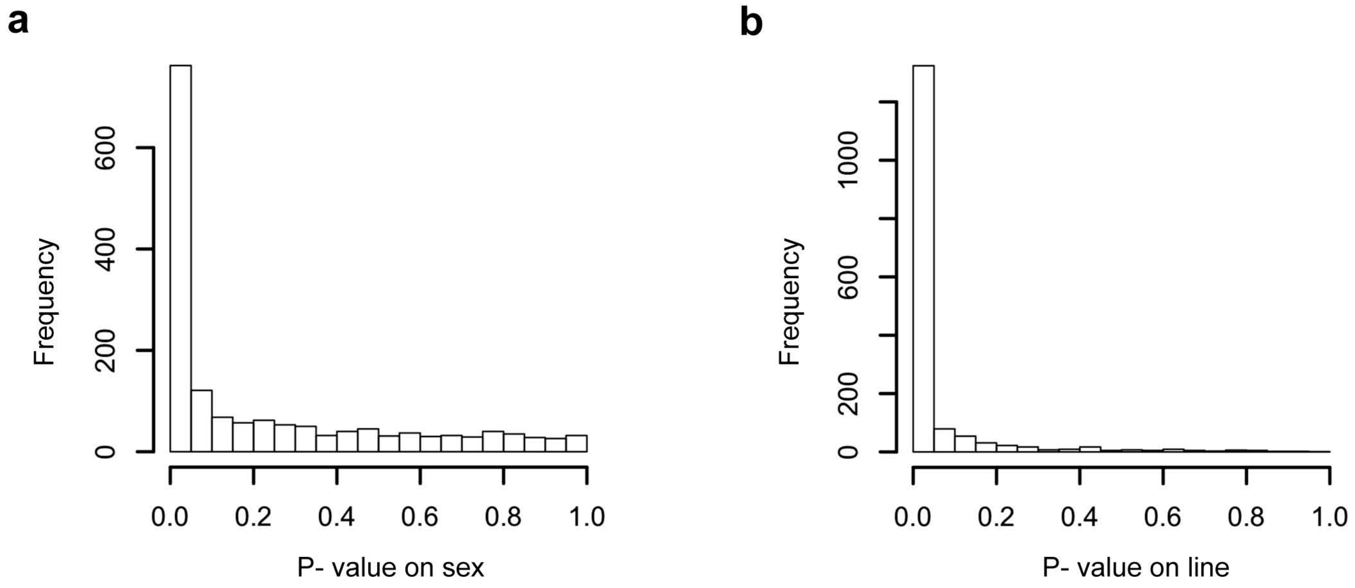

Supplementary Figure 2. P value distribution for the protein level difference between sexes and among lines

Significance of protein level variability between sexes (**a**) and among lines (**b**) was evaluated. 762 protein entries are significant for sex at p-value threshold 0.05, and 1,324 entries for lines. Combining, 1,394 (87%) protein entries showed a significant variability.

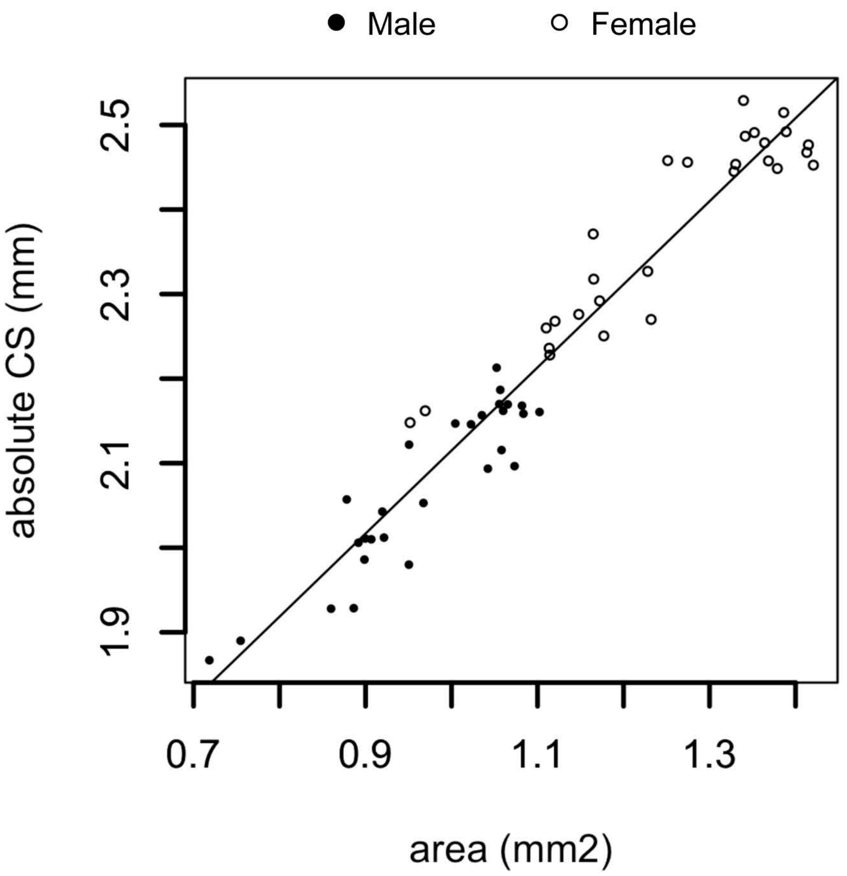

Supplementary Figure 3. Relationship between absolute CS and wing area
Absolute CS of samples is plotted against wing areas. The black and white circles indicate male and female samples, respectively. Note that absolute CS is proportional to wing area on the whole.

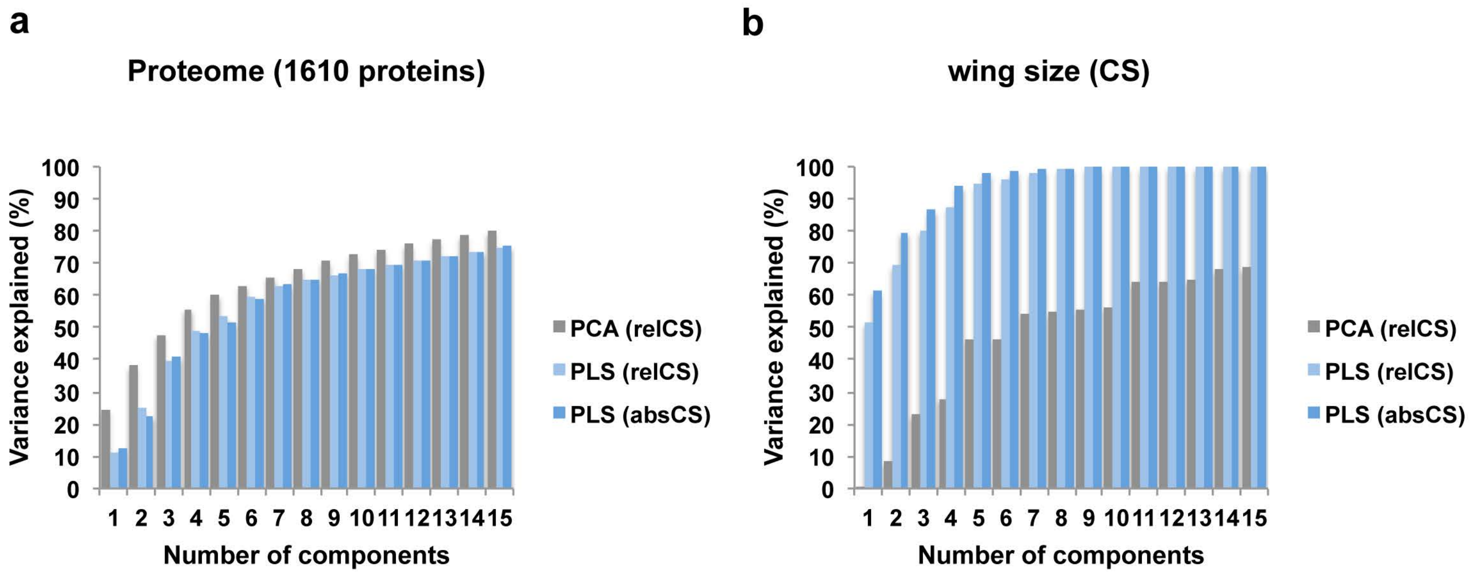

Supplementary Figure 4. Variances explained by PCA and PLS

PCA and PLS regressions were performed to explain variation of relative or absolute CS.

a, Variance of the whole proteome data matrix explained by the components of PCA or PLS. PCA components explain better the variance of proteome matrix than PLS does. **b**, Variance of wing size explained by the components of PCA or PLS. PLS components are superior to explain the variance of relative and absolute CSs.

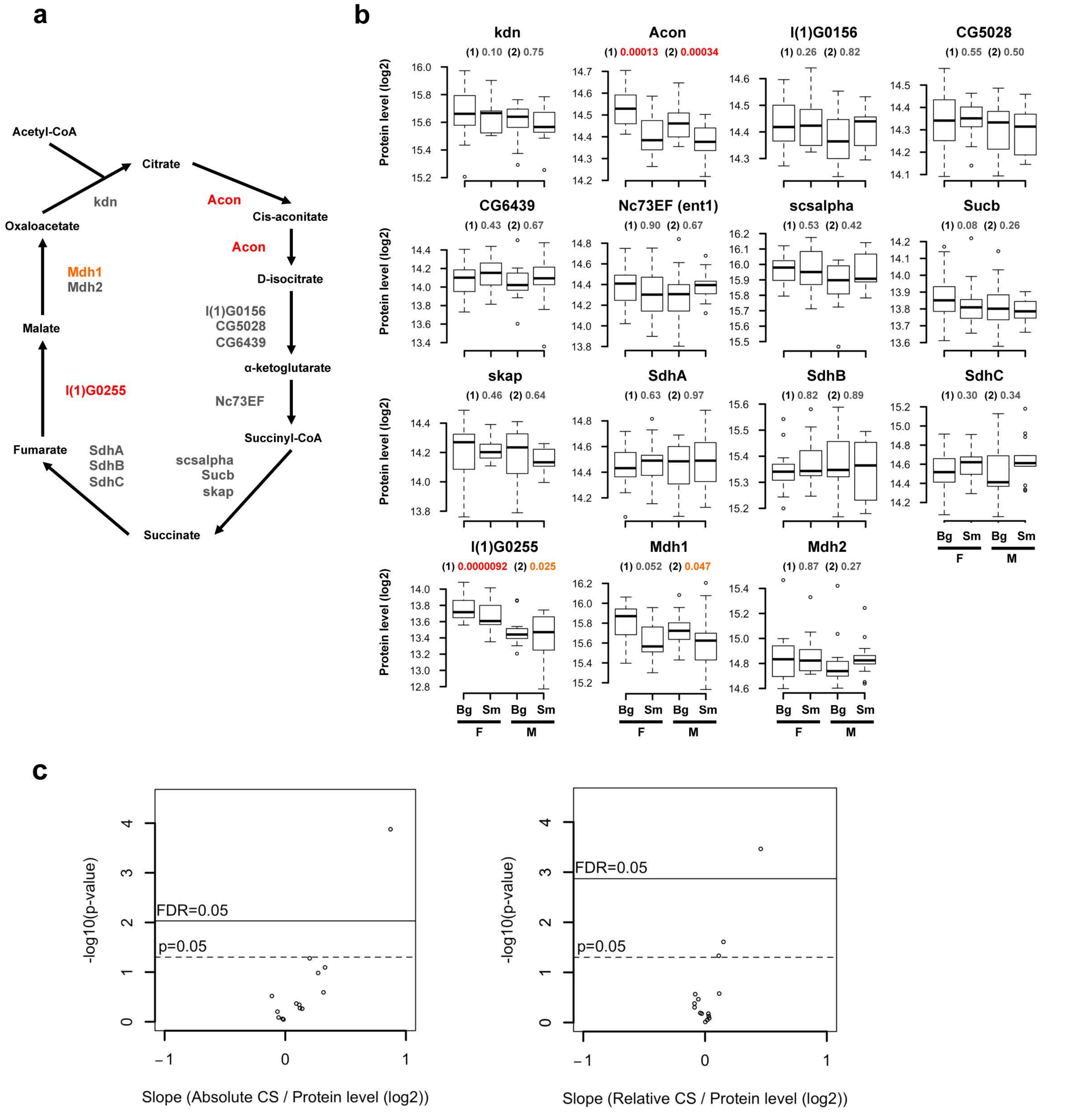

Supplementary Figure 5. Weak, positively-biased correlation of TCA cycle to wing size
a, Pathway map for TCA cycle. Proteins detected by SWATH-MS are shown. Proteins in red indicates association with either of absolute or relative CS at 5% FDR. Proteins in orange indicates association at nominal p-value <0.05. **b**, Protein levels of TCA cycle plotted against wing size for each sex. P-values are shown for association with absolute CS (1) and relative CS (2). Significance levels of association are indicated by color of p-values as in **a**. Bg: big wing samples, Sm: small wing samples, F: female, M: male. **c**, A weak, positive correlation of TCA cycle proteins to wing size. The p-values obtained in PWAS for absolute and relative CSs are plotted against slopes fitted in the model. The horizontal lines indicate significance thresholds as indicated.

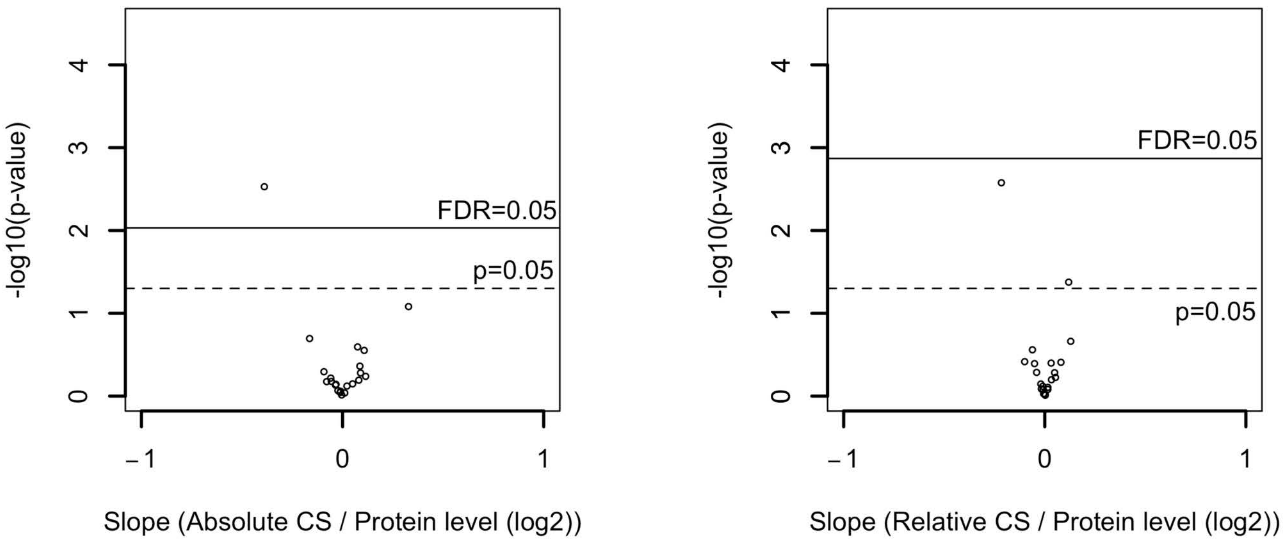

Supplementary Figure 6. Unbiased slope distribution of mitochondrial ribosomal proteins
The p-values obtained in PWAS for mitochondrial ribosomal proteins are plotted against slopes fitted in the model. The horizontal lines indicate significance thresholds as indicated.

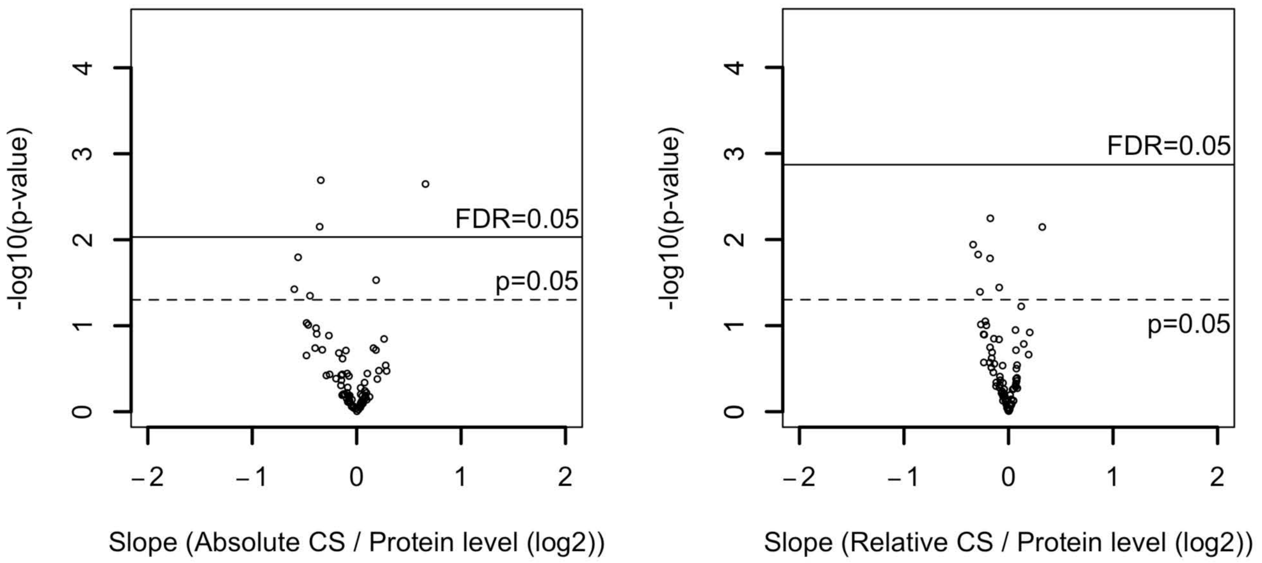

Supplementary Figure 7. Unbiased slope distribution of cytosolic ribosomal proteins
The p-values obtained in PWAS for cytosolic ribosomal proteins are plotted against slopes fitted in the model. The horizontal lines indicate significance thresholds as indicated.

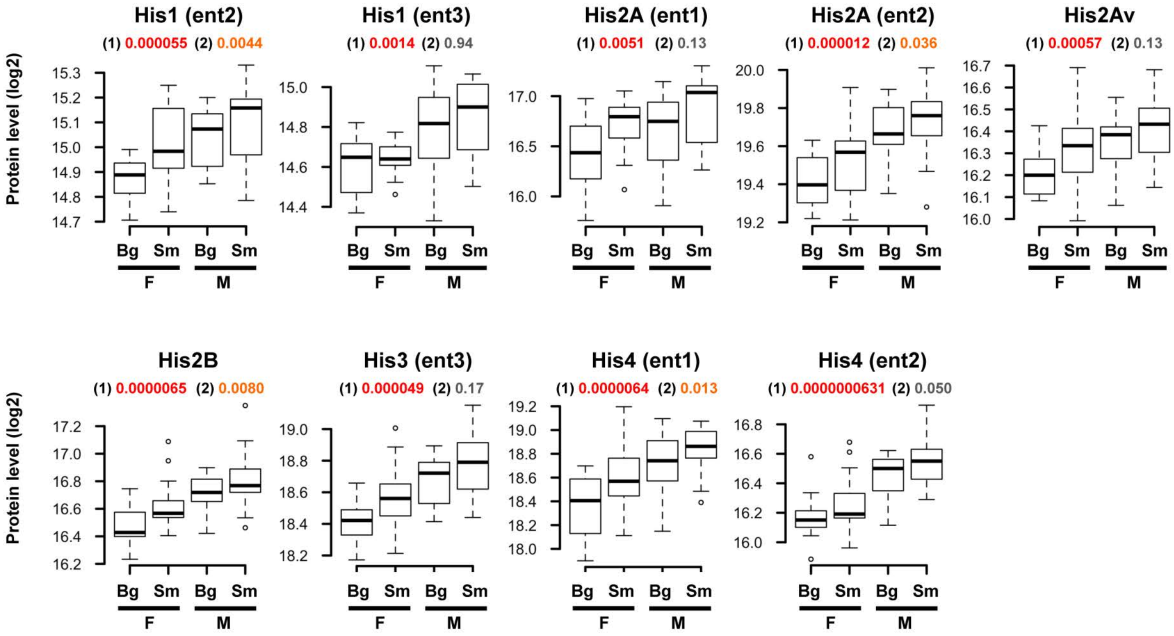

Supplementary Figure 8. Negative, systemic association of histone proteins with wing size
Levels of wing size-associated histone proteins are plotted against wing size for each sex. P-values are shown for association with absolute CS (1) and relative CS (2). Color of p-values indicates the significance levels of association (red: 5%FDR, orange: nominal p-value <0.05). Bg: big wing samples, Sm: small wing samples, F: female, M: male.

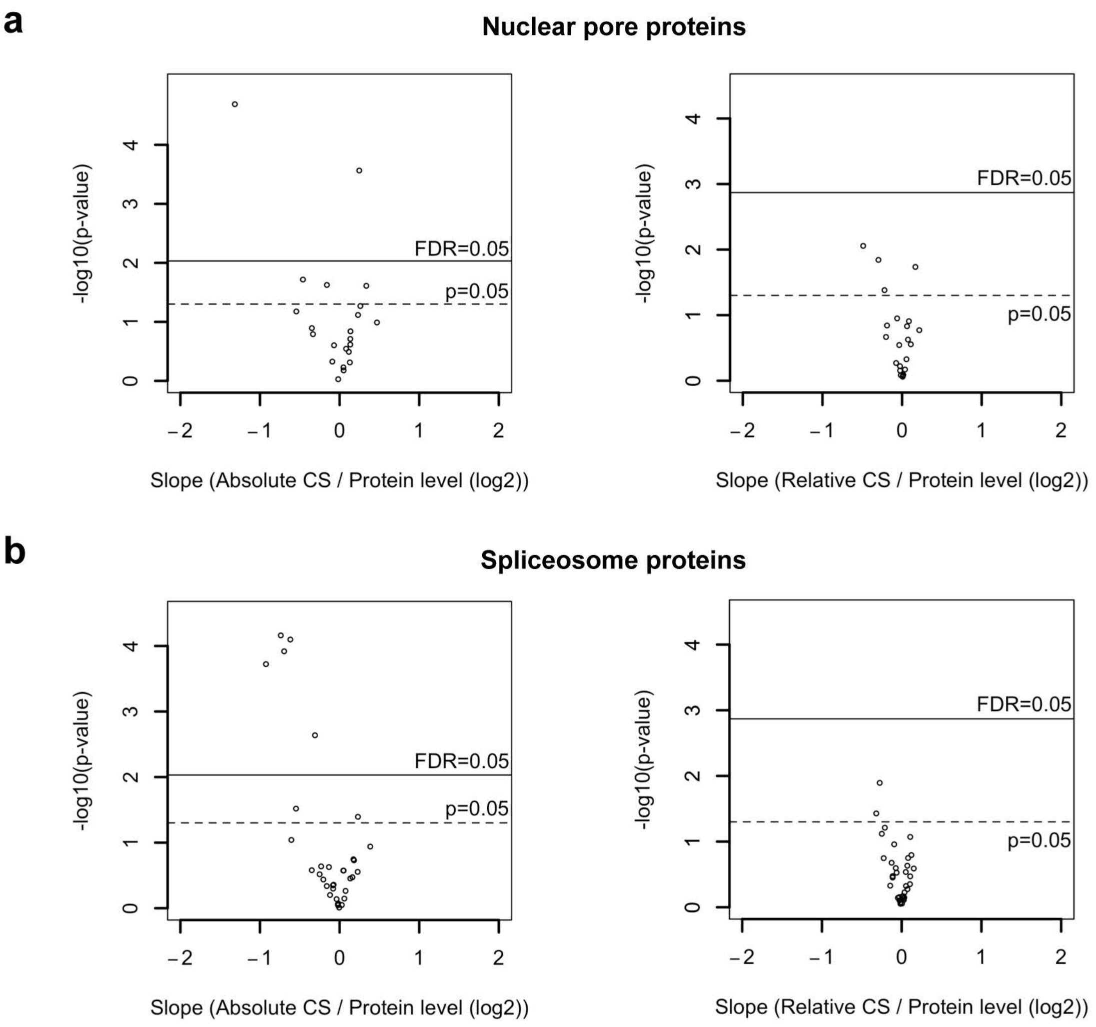

Supplementary Figure 9. Slope distributions for nuclear proteins

a and b, Unbiased slope distribution of the proteins from nuclear pore proteins and spliceosome. The p-values obtained in PWAS for absolute and relative CSs are plotted against slopes fitted in the model. The horizontal lines indicate significance thresholds as indicated.

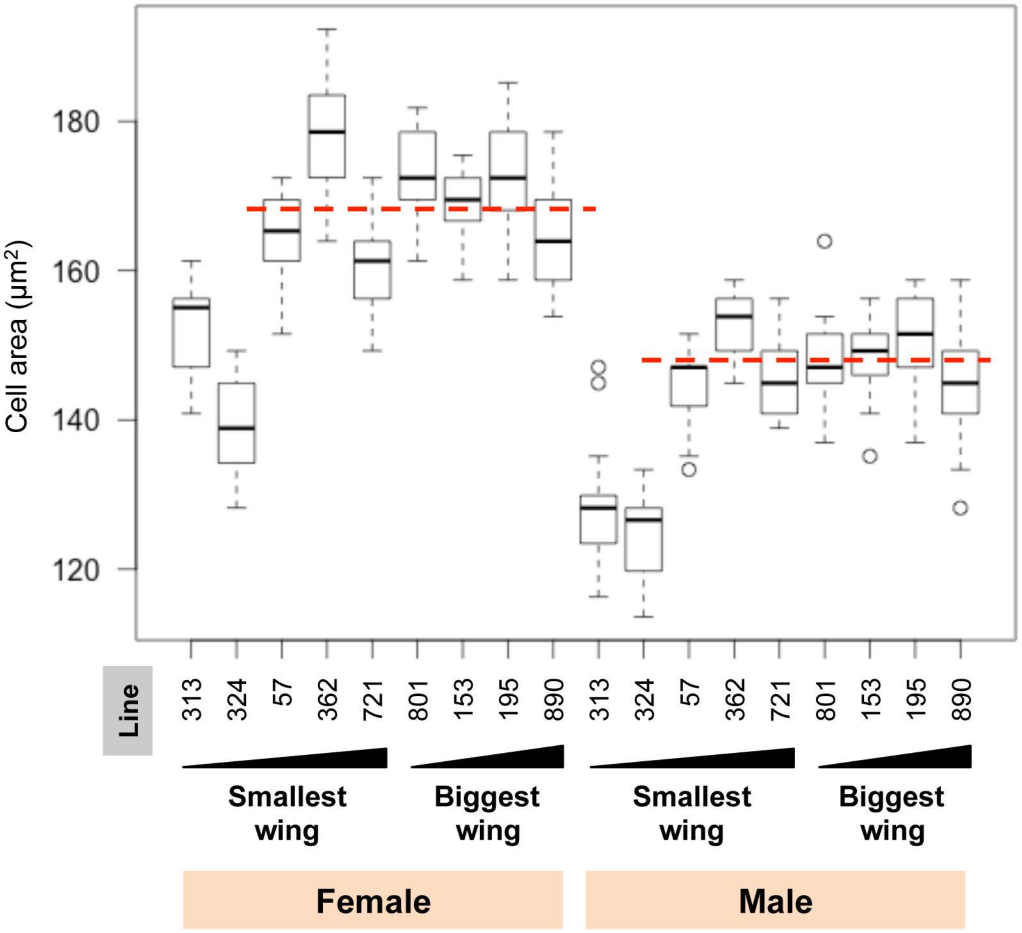

Supplementary Figure 10. Cell size in the wing

The average area of a single cell at the surface of the wing in the defined region (see Methods) are plotted for each sex of the lines with the 5 smallest wings and 4 biggest wings. Note that cell area is different between sexes but invariant within each sex (except the 2 smallest wing lines). The line IDs are shown.

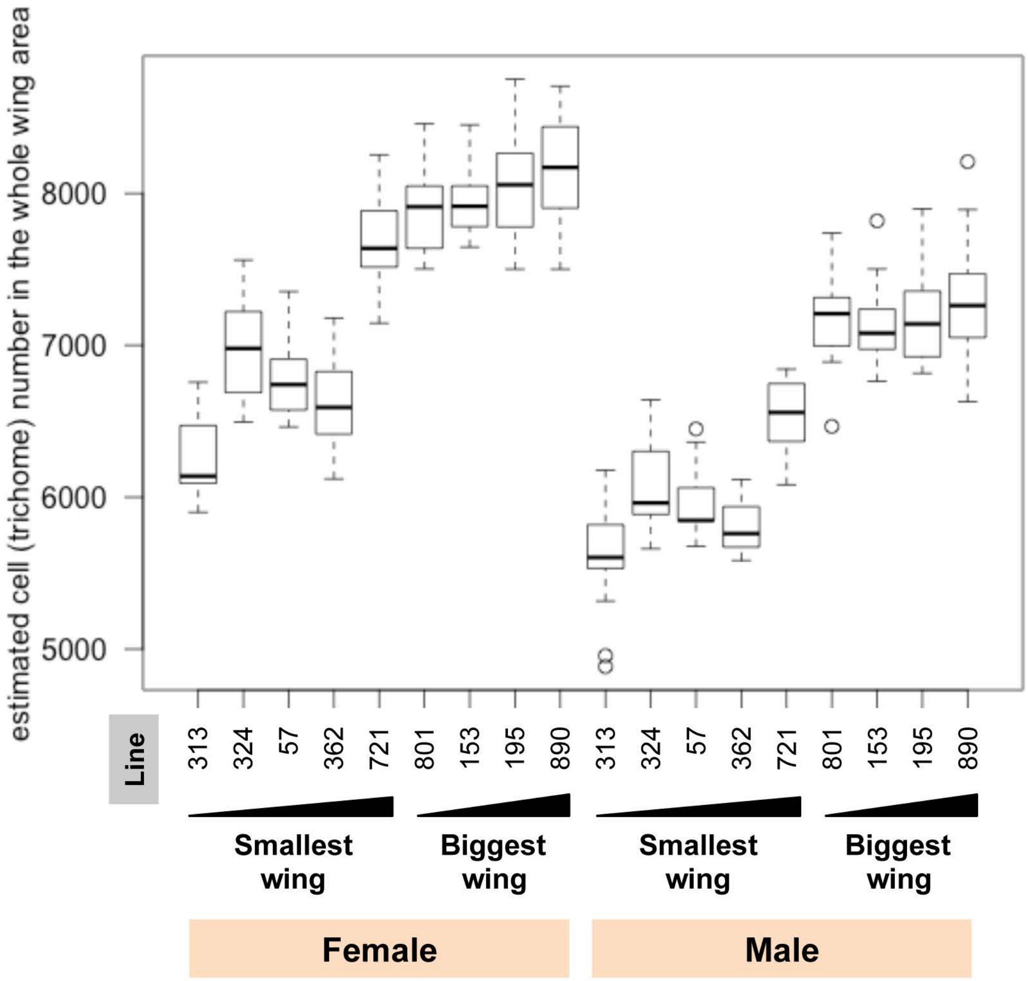

Supplementary Figure 11. Cell number in the wing

The total cell number in the whole wing area is estimated by dividing the whole wing area by the area of single cells. Note that the total cell numbers are distinct between the smallest and biggest wing lines within each sex. The line IDs are shown.

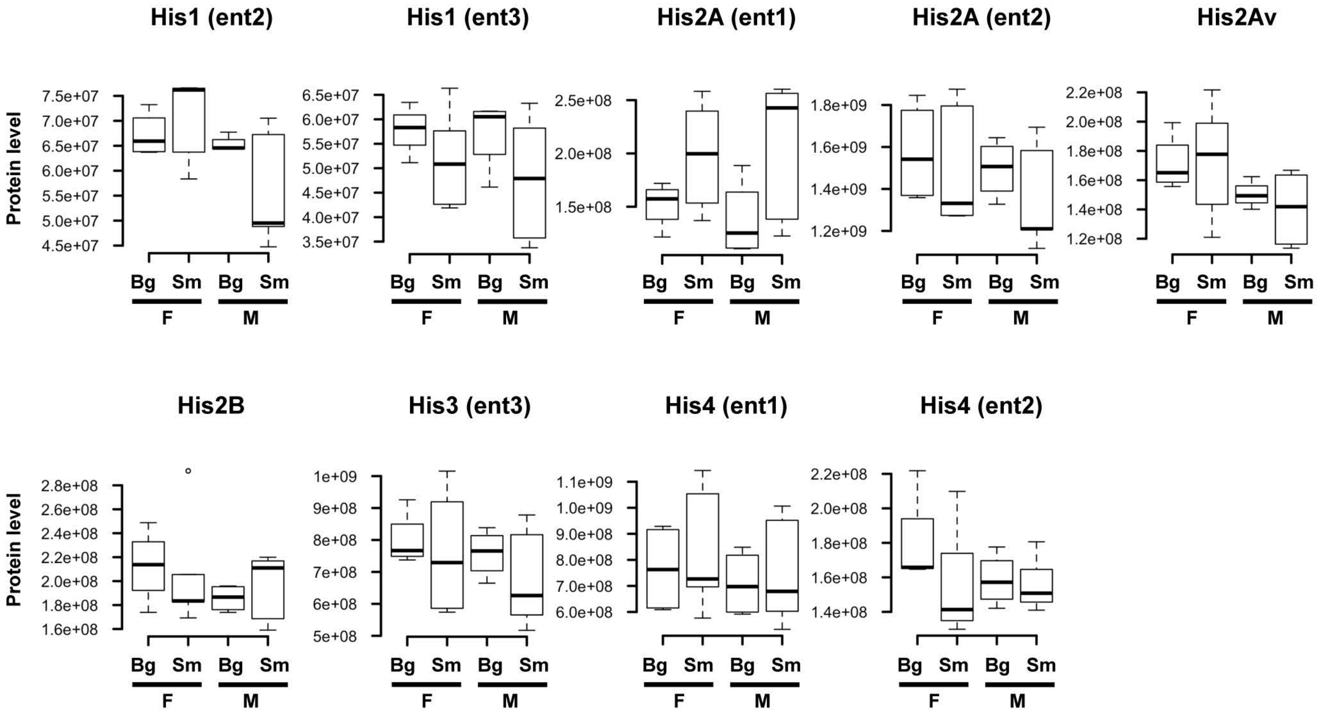

Supplementary Figure 12. Estimated histone protein levels per cell show no systemic correlation to wing size

The estimated approximate histone protein levels per cell are plotted against the 5 smallest wing lines and the 4 biggest wing lines separately for each sex. Note that the systemic, negative correlation of all histone proteins to wing size in Supplementary Fig. 8 is not observed in the plot.

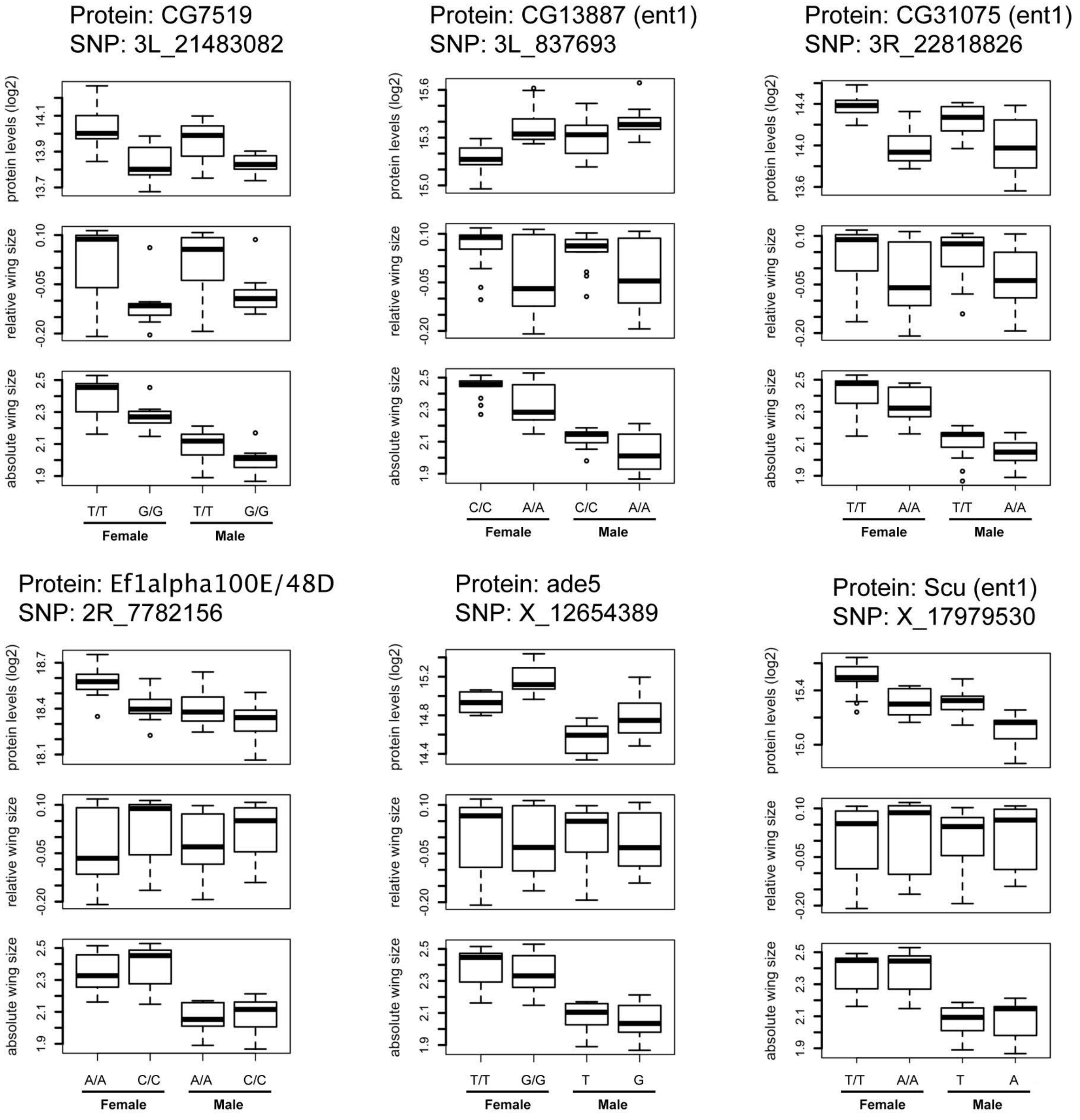

Supplementary Figure 13. Effect of female pQTLs on protein abundance and wing size
Protein and wing CS levels are plotted between the SNP variants at the pQTLs. Six protein entries passed the significance threshold at the corrected p-value of 0.01 in female. The pQTL with the smallest p-value for each protein entry was selected for the plots.

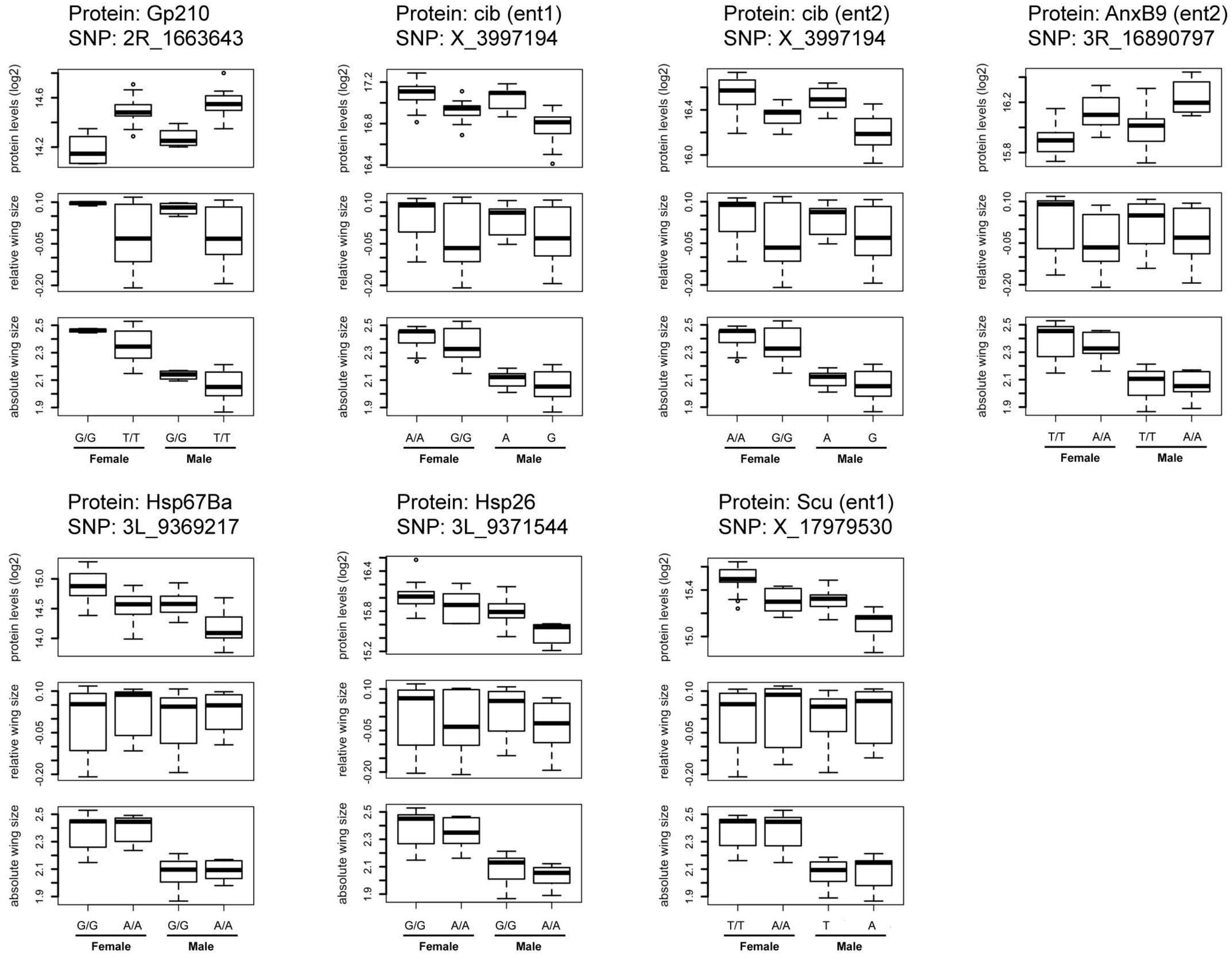

Supplementary Figure 14. Effect of male pQTLs on protein abundance and wing size
Protein and wing CS levels are plotted between the SNP variants at the pQTLs. Seven protein entries passed the significance threshold at the corrected p-value of 0.01 in male. The pQTL with the smallest p-value for each protein entry was selected for the plots.

Supplementary Table 1. PWAS results on mitochondrial respiratory chain complexes (1/2)

Gene.symbol	Activity	Absolute wing size		Relative wing size	
		P-value	FDR	P-value	FDR
**Complex I**					
l(3)neo18	NADH dehydrogenase (ubiquinone)	0.00058	0.007	0.0052	0.107
CG12203	NADH dehydrogenase (ubiquinone) 18 kDa subunit	0.019	0.083	0.168	0.542
NDUFA8	NADH dehydrogenase (ubiquinone) 19 kDa subunit	0.061	0.187	0.762	0.931
ND23	NADH dehydrogenase (ubiquinone) 23 kDa subunit	0.108	0.275	0.794	0.944
CG6020	NADH dehydrogenase (ubiquinone) 39 kDa subunit	0.259	0.487	0.450	0.797
ND42	NADH dehydrogenase (ubiquinone) 42 kDa subunit	0.020	0.086	0.153	0.524
CG1970	NADH dehydrogenase (ubiquinone) 49 kDa subunit	0.00014	0.003	0.0014	0.048
CG9140	NADH dehydrogenase (ubiquinone) 51 kDa subunit	0.967	0.979	0.657	0.888
CG10320	NADH dehydrogenase (ubiquinone) B12 subunit	0.832	0.917	0.825	0.951
CG3621	NADH dehydrogenase (ubiquinone) B14.5 A subunit	0.357	0.584	0.539	0.839
CG12400	NADH dehydrogenase (ubiquinone) B14.5 B subunit	0.533	0.733	0.698	0.900
CG12859	NADH dehydrogenase (ubiquinone) B15 subunit	0.0012	0.011	0.072	0.382
CG3446	NADH dehydrogenase (ubiquinone) B16.6 subunit	0.461	0.676	0.757	0.928
l(2)35Di	NADH dehydrogenase (ubiquinone) B17 subunit	0.034	0.128	0.038	0.276
CG9306	NADH dehydrogenase (ubiquinone) B22 subunit	0.396	0.618	0.497	0.821
CG32230	NADH dehydrogenase (ubiquinone) MLRQ subunit	0.449	0.667	0.032	0.255
Pdsw	NADH dehydrogenase (ubiquinone) PDSW subunit	0.047	0.158	0.208	0.598
ND75	NADH-ubiquinone oxidoreductase 75 kDa subunit	0.0025	0.018	0.146	0.514
mt:ND3	NADH-ubiquinone oxidoreductase chain 3	0.128	0.309	0.716	0.910
**Complex II**					
SdhA	Succinate dehydrogenase, subunit A (flavoprotein)	0.626	0.802	0.974	0.991
SdhB	Succinate dehydrogenase, subunit B (iron-sulfur)	0.820	0.911	0.891	0.970
SdhC	Succinate dehydrogenase, subunit C	0.303	0.531	0.344	0.719
**Complex III**					
RFeSP	Cytochrome b-c1 complex subunit Rieske	0.139	0.327	0.362	0.733
CG3560	Ubiquinol-cytochrome c reductase 14 kDa subunit	0.036	0.131	0.070	0.381
CG3731	Ubiquinol-cytochrome c reductase core protein 1	0.319	0.547	0.191	0.572
CG4169	Ubiquinol-cytochrome c reductase core protein 2	0.322	0.550	0.411	0.766

Supplementary Table 1. PWAS results on mitochondrial respiratory chain complexes (2/2)

		Absolute wing size		Relative wing size	
**Gene.symbol**	**Activity**	**P-value**	**FDR**	**P-value**	**FDR**
**Complex IV**					
mt:CoII	Cytochrome c oxidase subunit 2	0.133	0.318	0.763	0.931
CoIV	Cytochrome c oxidase subunit 4	0.210	0.423	0.607	0.870
CoVa	Cytochrome c oxidase subunit 5A	0.523	0.726	0.551	0.845
CoVb	Cytochrome c oxidase subunit 5B	0.458	0.674	0.637	0.884
CoVIb	Cytochrome c oxidase subunit 6B	0.291	0.518	0.966	0.991
cype	Cytochrome c oxidase subunit 6C	0.477	0.688	0.778	0.937
CG9603	Cytochrome c oxidase subunit 7A	0.531	0.732	0.449	0.796
levy	Cytochrome c oxidase, subunit VIa	0.266	0.491	0.548	0.844
**Complex V (ATP synthase)**					
mt:ATPase8	ATP synthase protein 8	0.355	0.582	0.947	0.982
blw	ATP synthase subunit alpha	0.523	0.726	0.862	0.962
Oscp	ATP synthase, oligomycin sensitivity conferring protein	0.065	0.197	0.326	0.705
ATPsyn-b	ATP synthase, subunit B	0.687	0.830	0.841	0.953
ATPsyn-d	ATP synthase, subunit D	0.926	0.965	0.603	0.870
CG3321	ATP synthase, subunit E	0.138	0.327	0.234	0.625
CG4692	ATP synthase, subunit F	0.574	0.760	0.649	0.885
ATPsyn-beta	ATP synthase, beta subunit	0.604	0.785	0.960	0.990
ATPsyn-gamma	ATP synthase, gamma subunit	0.479	0.689	0.480	0.813
l(1)G0230	ATP synthase, delta/epsilon subunit	0.048	0.160	0.284	0.667
ATPsyn-Cf6	ATPase, F0 complex, subunit F6	0.265	0.490	0.677	0.897
l(2)06225	ATPase, F0 complex, subunit G	0.953	0.972	0.895	0.971
sun	ATPase, F1 complex, epsilon subunit	0.501	0.708	0.746	0.922
**Others**					
Etf-QO	Electron transfer flavoprotein-ubiquinone oxidoreductase	0.054	0.172	0.353	0.726
wal	Electron transfer flavoprotein, alpha subunit	0.669	0.823	0.587	0.861
CG7834	Electron transfer flavoprotein, beta subunit	0.614	0.791	0.812	0.950
mt:Cyt-b	Cytochrome b	0.288	0.516	0.126	0.491
Cyt-c-p	Cytochrome c-2	0.686	0.829	0.106	0.471
CG4769	Cytochrome c1	0.115	0.287	0.013	0.163

Supplementary Note 1

In silico search parameters:

```
MZXML = HEBHARDT_L130831_002.mzXML, HEBHARDT_L130831_004.mzXML,
HEBHARDT_L130831_001.mzXML, HEBHARDT_L130831_003.mzXML
RUNTANDEM = True
QUIET = False
DBASENAME = dmel-all-translation-r5.52
XTANDEM_SCORE = default
DB_SOURCE = BioDB
OUTEXPERIMENT = E20130710_1039_GSVDXK_0
experiment-code = E1309031318
PRECMASSUNIT = ppm
LOG_LEVEL = DEBUG
VARIABLE_MODS = Oxidation (M)
MISSEDCLEAVAGE = 1
FRAGMASSERR = 0.1
DBASE = dmel-all-translation-r5.52.fasta
DSSCLIENT = getmsdata
DATABASE_VERSION = 20130901
GENERATOR_CHECKSUM = 1
PEPTIDEFDR = 0.01
PARAM_IDX = 0
WORKFLOW = TPP_2013-08-29-150752_20130903131654886 imsbtools/20130215
applicake@1483
DECOY_STRING = DECOY_
PROJECT = WINGS-PROTEOMICS
STATIC_MODS = Carbamidomethyl (C)
DATABASE_PACKAGE = ex_fb
DSSKEYS = ""
XINTERACT_ARGS = -dDECOY_ -p0 -OAPdlIw (dummy)
PEPXMLS = InterProphet.pep.xml,
DATABASE_DB = dmel-all-translation-r5.52
FRAGMASSUNIT = Da
COMMENT = WINGS_1st_plus_iRT
RUNPETUNIA = full
IPROBABILITY = 0.8400
ENZYME = Trypsin
THREADS = 8
RESULT_FILE = ""
IPROPHET_ARGS = MINPROB=0
FAILURE_TOLERANT = False
```

Spectral library generation:

```
TSV_REMOVE_DUPLICATES = True
TSV_PRECISION = 0.05
TSV_SERIES = b;y
RSQ_THRESHOLD = 0.95
TSV_GAIN = -17;-18;-64
TSV_ION_LIMITS = 3-5
HASSPLIB = true
PRECURSORLEVEL = False
PEPTIDEFDR = 0.01
```

WORKFLOW = LibraryCreation_2013-08-29-150809_20130903153447041
imsbtools/20130215 applicake@1483
CONSENSUS_TYPE = Best replicate
VERSION = 20130903160855
TSV_CHARGE = 1;2;3
DESCRIPTION = wing-disc-plus-iRT
RUNRT = True
NAME = Wing-discs-SWATH-lib-iRT
TSV_MASS_LIMITS = 400-2000
APPLYCHAUVENET = False
MS_TYPE = CID-QTOF
SPECTRALEVEL = False
TSV_EXACT = True
RTKIT = ""

SWATH-search:
MIN_RSQ = 0.95
ALIGNER_DSCORE_CUTOFF = 0.5
WORKFLOW = openswath_requant_2014-05-26-161908_20141128135140327
imsbtools/20140808 applicake@09564a8 msproteomicstools@590
openms@7c408dd
WINDOW_UNIT = Thomson
ALIGNER_TARGETFDR = 0.01
MIN_UPPER_EDGE_DIST = 1
ALIGNER_MAX_RT_DIFF = 30
IRTTRAML = /cluster/apps/imsbtools/stable/files/hroest_DIA_iRT.TraML
MPR_MAINVAR = xx_swath_prelim_score
COMMENT = 119-PLUS-REQUANT
MIN_COVERAGE = 0.6
RT_EXTRACTION_WINDOW = 600
MPR_VARS = bseries_score elution_model_fit_score intensity_score
isotope_correlation_score isotope_overlap_score library_corr
library_rmsd log_sn_score massdev_score massdev_score_weighted
norm_rt_score xcorr_coelution xcorr_coelution_weighted xcorr_shape
xcorr_shape_weighted yseries_score
ALIGNER_FRACSELECTED = 0
EXTRACTION_WINDOW = 0.05
MPR_NUM_XVAL = 10
ALIGNER_METHOD = best_overall
DO_CHROMML_REQUANT = true
